# Clinical Perspectives on Using Remote Measurement Technology in Assessing Epilepsy, Multiple Sclerosis, and Depression: Delphi Study

**DOI:** 10.2196/41439

**Published:** 2023-04-25

**Authors:** Jacob A Andrews, Michael P Craven, Boliang Guo, Janice Weyer, Simon Lees, Spyridon I Zormpas, Sarah E Thorpe, Julie Devonshire, Victoria San Antonio-Arce, William P Whitehouse, Jessica Julie, Sam Malins, Alexander Hammers, Andreas Reif, Henricus G Ruhe, Federico Durbano, Stefano Barlati, Arjune Sen, Jette L Frederiksen, Alessandra Martinelli, Antonio Callen, Joan Torras-Borrell, Nuria Berrocal-Izquierdo, Ana Zabalza, Richard Morriss, Chris Hollis, The RADAR-CNS Consortium

**Affiliations:** 1 National Institute for Health and Care Research MindTech MedTech Co-operative University of Nottingham Nottingham United Kingdom; 2 Academic Unit of Mental Health and Clinical Neurosciences School of Medicine University of Nottingham Nottingham United Kingdom; 3 Human Factors Research Group Faculty of Engineering University of Nottingham Nottingham United Kingdom; 4 National Institute for Health and Care Research Applied Research Collaboration East Midlands School of Medicine University of Nottingham Nottingham United Kingdom; 5 Patient Advisory Board RADAR-CNS Kings College London London United Kingdom; 6 Freiburg Epilepsy Center, Medical Center Faculty of Medicine University of Freiburg Freiburg Germany; 7 Division of Child Health, Obstetrics and Gynaecology School of Medicine University of Nottingham Nottingham United Kingdom; 8 National Institute for Health and Care Research Biomedical Research Centre for Mental Health Kings College London London United Kingdom; 9 Nottinghamshire Healthcare National Health Service Foundation Trust Nottingham United Kingdom; 10 School of Biomedical Engineering and Imaging Sciences King’s College London London United Kingdom; 11 Department of Psychiatry, Psychosomatic Medicine and Psychotherapy Goethe University Frankfurt am Main Germany; 12 Department of Psychiatry Radboud University Medical Center Nijmegen Netherlands; 13 Donders Institute for Brain, Cognition and Behavior Radboud University Nijmegen Netherlands; 14 Department of Mental Health and Addictions Aziende Socio Sanitarie Territoriali Melegnano e della Martesana Lombardy Italy; 15 Department of Clinical and Experimental Sciences University of Brescia Brescia Italy; 16 Oxford Epilepsy Research Group Nuffield Department of Clinical Neurosciences University of Oxford Oxford United Kingdom; 17 Department of Neurology Rigshospitalet Glostrup University of Copenhagen Copenhagen Denmark; 18 Istituto di Ricovero e Cura a Carattere Scientifico Istituto Centro San Giovanni di Dio Fatebenefratelli Brescia Italy; 19 Department of Neurology Parc Sanitari Sant Joan de Déu Barcelona Spain; 20 Centre d'Atenció Primària Sant Llàtzer Consorci Sanitari de Terrassa Barcelona Spain; 21 Parc Sanitari Sant Joan de Deu Barcelona Spain; 22 Servei de Neurologia-Neuroimmunologia Centre d’Esclerosi Múltiple de Catalunya (Cemcat) Vall d’Hebron Hospital Universitari Barcelona Spain; 23 Vall d’Hebron Institut de Recerca Barcelona Spain; 24 Kings College London London United Kingdom

**Keywords:** mobile health, mHealth, wearable, wearable technology, smartphone, use case, implementation, epilepsy, multiple sclerosis, MS, depression, depressive disorder, Delphi, remote measurement technology, RMT, central nervous system, nervous system disorder, neurology, neurological disorder, expert panel, expert opinion, perspective, mobile phone

## Abstract

**Background:**

Multiple sclerosis (MS), epilepsy, and depression are chronic central nervous system conditions in which remote measurement technology (RMT) may offer benefits compared with usual assessment. We previously worked with clinicians, patients, and researchers to develop 13 use cases for RMT: 5 in epilepsy (seizure alert, seizure counting, risk scoring, triage support, and trend analysis), 3 in MS (detecting silent progression, detecting depression in MS, and donating data to a biobank), and 5 in depression (detecting trends, reviewing treatment, self-management, comorbid monitoring, and carer alert).

**Objective:**

In this study, we aimed to evaluate the use cases and related implementation issues with an expert panel of clinicians external to our project consortium.

**Methods:**

We used a Delphi exercise to validate the use cases and suggest a prioritization among them and to ascertain the importance of a variety of implementation issues related to RMT. The expert panel included clinicians from across Europe who were external to the project consortium. The study had 2 survey rounds (n=23 and n=17) and a follow-up interview round (n=9). Data were analyzed for consensus between participants and for stability between survey rounds. The interviews explored the reasons for answers given in the survey.

**Results:**

The findings showed high stability between rounds on questions related to specific use cases but lower stability on questions relating to wider issues around the implementation of RMT. Overall, questions on wider issues also had less consensus. All 5 use cases for epilepsy (seizure alert, seizure counting, risk scoring, triage support, and trend analysis) were considered beneficial, with consensus among participants above the a priori threshold for most questions, although use case 3 (risk scoring) was considered less likely to facilitate or catalyze care. There was very little consensus on the benefits of the use cases in MS, although this may have resulted from a higher dropout rate of MS clinicians (50%). Participants agreed that there would be benefits for all 5 of the depression use cases, although fewer questions on use case 4 (triage support) reached consensus agreement than for depression use cases 1 (detecting trends), 2 (reviewing treatment), 3 (self-management), and 5 (carer alert). The qualitative analysis revealed further insights into each use case and generated 8 themes on practical issues related to implementation.

**Conclusions:**

Overall, these findings inform the prioritization of use cases for RMT that could be developed in future work, which may include clinical trials, cost-effectiveness studies, and the commercial development of RMT products and services. Priorities for further development include the use of RMT to provide more accurate records of symptoms and treatment response than is currently possible and to provide data that could help inform patient triage and generate timely alerts for patients and carers.

## Introduction

### Background

Digital and mobile health technologies, including smartphone-based monitoring and wearable devices, have a wide range of applications in clinical practice [1-3]. A clinical “use case” describes how a technology can be implemented in a clinical context, including the expected benefit and expected beneficiary. Clinical use cases are essential for determining the outcomes to be used in trials evaluating effectiveness as well as for obtaining regulatory approvals and explaining the benefits of a health care technology to potential funders and patients. The adoption and scaling of novel technologies in health care are dependent on a well-defined use case with a clearly defined problem to be addressed [4]. The inclusion of clinicians in the development of such technologies is known to be important for successful implementation, as it ensures the appropriateness of technology for the specific requirements of patients and the health care system [5].

Remote Assessment of Disease and Relapse–Central Nervous System (RADAR-CNS) was a 6-year project to understand the feasibility and acceptability of using remote measurement technology (RMT) to collect health-relevant data from individuals living with epilepsy, multiple sclerosis (MS), or depression [6]. The project was a collaboration across 6 European countries (Denmark, Germany, Italy, the Netherlands, Spain, and the United Kingdom) and has involved the development of a bespoke, open-source platform RADAR-base. The platform collates data from commercially available Fitbit smart watches measuring activity, heart rate, and heart rate variability; the Empatica E4 wrist-worn epilepsy seizure detection device; Bittium Faros accelerometer and electrocardiogram Holter devices; and bespoke apps for passive sensing and active collection of user-entered data (THINC-it) [7]. We refer to the combination of the platform, the apps, and the commercial devices as the RADAR-CNS RMT system. Observational studies have been conducted to establish the feasibility and acceptability of collecting data from individuals living with MS, epilepsy, or depression using these sensors, apps and platform to develop new predictive algorithms based on the data set [8-10]. Patient involvement has been conducted throughout the program, and patient focus groups and other involvement studies have been conducted in multiple European countries to elicit patient views and inform the RMT under development [10-13].

The aim of this study was to specify priority use cases for RMT in 3 central nervous system disorders (epilepsy, MS, and depression). An initial set of 13 use cases were developed through discussion with health care professionals (HCPs) and researchers working in each of the 3 clinical work packages within the project. The development of these use cases considered the fit to the target population, the potential for a positive impact on the health and safety of patients, whether the use case would offer an improvement on current methods, and the existence of prior evidence to support the use case. These were also informed by our prior work, which included: a small-scale survey with patient advisers, HCPs, and researchers [14]; in-depth interviews with HCPs [15]; and a large-scale survey of 1006 clinicians on the current and potential use of RMT and apps in clinical practice [16] and the potential value of remote measurement data [17]. The Delphi study then sought to prioritize among the 13 use cases (5 in epilepsy, 3 in MS, and 5 in depression) to determine which of these would be most practicable and useful in the eyes of the expert clinician panel, who were outside of the consortium and so offered a more objective point of view. The number of use cases included in the study was considered to be manageable without overburdening participants.

The use cases were also presented to the RADAR-CNS Patient Advisory Board (PAB) to seek further input ahead of this study in a short consultation via Microsoft Teams, with diagrams and descriptions of use cases provided by email in advance. The RADAR-CNS PAB includes members living with each of the 3 conditions from multiple countries across Europe. Illustrations of the 13 use cases are included in Multimedia Appendix 1.

The final use cases for epilepsy are as follows:

1. Seizure alert: enabling real-time seizure warnings to patients and carers.
2. Seizure counting: improving detection of different types of seizures to enable more accurate overall seizure records.
3. Risk scoring: detecting cycles of seizure occurrence to reveal risk levels at different times.
4. Triage support: enhancing patient triage based on RMT data submitted wirelessly to patient record systems.
5. Trend analysis: reliably detecting a change in the number of seizures that a patient has over a specified period.

The final use cases for MS are as follows:

1. Detecting silent progression: making use of more granular measurements to detect otherwise invisible markers of progression, enabling patients to evidence changes they experience.
2. Detecting depression: identifying markers of depression in the first year after MS diagnosis.
3. Data donation: automatic collection and storage of patient data in biobanks or mega-databases.

The final use cases for depression are as follows:

1. Detecting trends: detailed symptom tracking and aggregation of multiple types of data.
2. Treatment review: measuring adherence to cognitive behavioral therapy or other treatment regimens and treatment response.
3. Self-management: monitoring and providing nudges to a patient to improve their condition.
4. Comorbid monitoring: detecting depression in patients with chronic physical health conditions.
5. Carer Alert: providing an alert to a carer or relative when a person with depression is in a period of very low activity.

### Aims and Objectives

The aim of this work was to prioritize the use cases with the potential for the greatest benefit for further development according to the views of HCPs external to the project. We sought to establish the type of benefit that each might offer according to a medical device design framework [18]. We also aimed to explore further related issues:

1. Acceptability of the level of burden (clinical time) required to apply RMT in practice.
2. Acceptability of the amount of data that would be generated by RMT.
3. The extent of required technical support for clinicians to make the best use of RMT.
4. Preferred mode of training or technical support for clinicians.
5. The extent of required technical support for patients to make the best use of RMT.

We aimed to seek a consensus in these areas, where prior work has shown that there is a disagreement between HCPs. In addition, we aimed to determine which prior known concerns about the use of RMT in clinical practice would actually prevent or discourage HCPs from using RMT with patients. As we recruited an international sample, we were also interested in exploring how the potential implementation of RMT might differ between countries.

## Methods

### Overview

The Delphi methodology has been widely used in health care research to gather expert opinions [19-21]. Key characteristics of the Delphi method include consultation of experts, elements of iteration and feedback to participants to enable a form of communication between them, and statistical methods used to summarize group responses to ensure the robustness of analyses [22]. Delphi studies can be used without face-to-face contact while still enabling the gathering of group opinions, which is of benefit when those whose opinions are required have busy schedules (eg, in clinical settings) or may be located across multiple countries [23-25], as is the case in this study. There were also obvious benefits to this approach during the COVID-19 pandemic.

Delphi studies feature multiple survey rounds, with feedback given to the participants between each. For example, Murphy et al [21] used a 3-round model to gather views and opinions on the potential of digital tools for mental health in the United Kingdom. The first round of a Delphi study typically asks open-ended questions, and these are used to generate closed questions for a survey in the second round, often with Likert-style responses. In the third round (if there is one), participants review the summarized results from the prior round and are then able to change their responses if required [26]. This results in either greater consensus among the groups or sustained disagreement, both of which are of interest [25]. Other models may omit the first qualitative round [19] and may include follow-up interviews after the final survey round [27].

### Procedure

#### Overview

**Figure 1:**
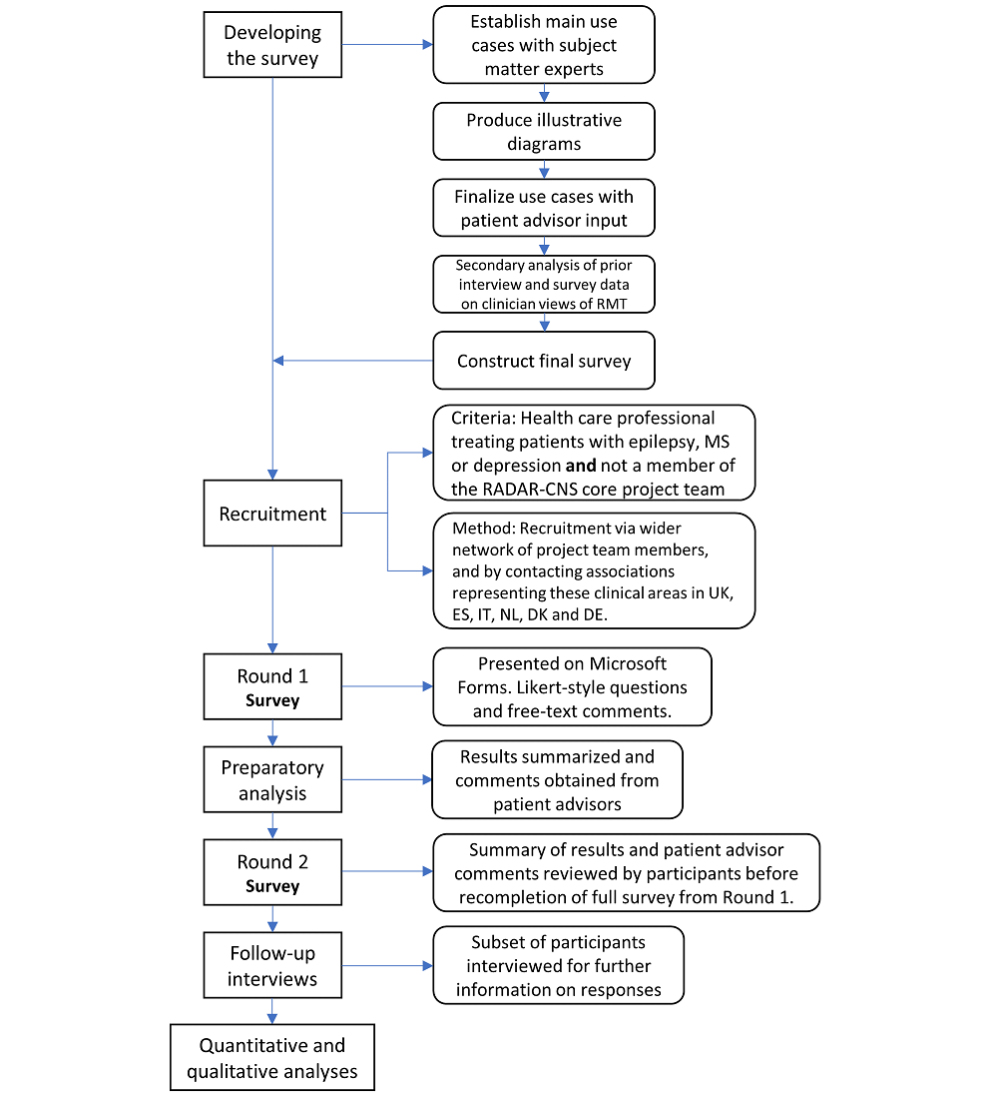
Flowchart showing study process. DE: Germany; DK: Denmark; ES: Spain; IT: Italy; MS: multiple sclerosis; NL: Netherlands; RADAR-CNS: Remote Assessment of Disease and Relapse–Central Nervous System; RMT: remote measurement technology; UK: United Kingdom.

This study adopted the Delphi methodology for the context of RADAR-CNS. The study procedure is summarized in Figure 1. As the project had already canvassed opinions from HCPs in surveys and interviews, we replaced the first qualitative round with a reanalysis of our existing data to generate the survey for use in this study. It is recommended that Delphi surveys be completed within 30 minutes [25]. Thus, we used diagrams of use cases (included in Multimedia Appendix 1) to aid rapid comprehension and to permit engagement with the ideas presented.

There was a gap of 3 months between the dissemination of the first survey round and the dissemination of the second survey round. After the first round, the research team gathered responses and produced graphs and tables to form a Summary of Results document. In a further adaptation to the traditional Delphi methodology, this Summary of Results was presented to the RADAR-CNS PAB to request commentary on clinicians’ responses. Patients who reviewed the round 1 responses consisted of 2 people: 1 living with epilepsy (male) and 1 living with MS (female). Unfortunately, the members of the PAB living with depression did not respond to requests to provide comments. Patients were sent a summary of round 1 results in graphs, tables, and free-text comments, along with a short video explaining what was expected from them. They wrote their comments in a word document or email and returned them to the first author.

**Figure 2:**
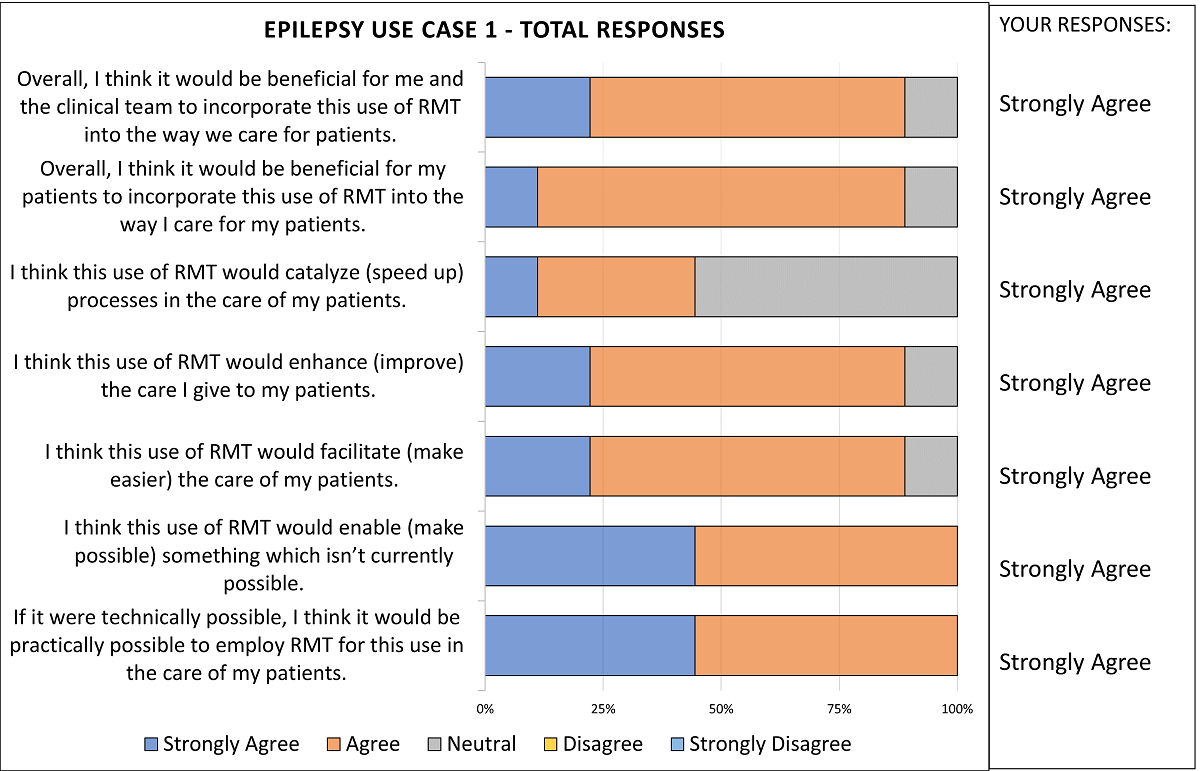
An example showing the style of feedback provided to Delphi panel members after round 1 and showing how individual responses were combined with group responses in the Summary of Results. RMT: remote measurement technology.

For round 2, HCP participants received the Summary of Results, which incorporated patient comments, together with a link to the round 2 survey for completion, and were instructed to review the Summary of Results before completing the second round. The Summary of Results was personalized to each participant, with their own responses indicated next to graphs showing summary responses (Figure 2). Graphs were used to provide an “at-a-glance” overview of the results for quick interpretation. Free-text comments from Delphi experts and from patients were provided in boxes below the graphs for participants to review.

Delphi studies have been criticized for their closed nature, which prevents discussion of the results [26]. This study sought to overcome this criticism by including follow-up interviews to discuss the results with individual participants. Therefore, we used a mixed method sequential explanatory approach to collect our data. Figure 1 shows a flowchart summarizing the process.

#### Recruitment

HCPs were recruited from multiple countries where RADAR is active via multiple routes. The main method of recruitment was by clinical academics within the RADAR-CNS consortium disseminating recruitment materials to their clinical academic colleagues. In addition, we contacted specialist associations representing clinicians treating each of the 3 conditions across the 6 European countries of the consortium (the United Kingdom, Germany, Italy, Spain, the Netherlands, and Denmark). We also contacted prior research participants external to the consortium and contacted clinicians who had previously expressed interest in the RADAR-CNS project.

#### Inclusion Criteria

We required participants to be experts in the area by virtue of their experience working in the clinical care of people with epilepsy, MS, or depression. We specified that participants should not be members of the RADAR-CNS consortium to gain an external view of the potential of the technology in clinical practice.

#### Survey Design

The survey was composed of 4 main sections: demographics, use case evaluation, questions related to the implementation of RMT, and rating of concerns. The full survey is included in Multimedia Appendix 2.

There were 3 separate use case evaluation sections, one for each clinical condition. The epilepsy section evaluated 5 use cases, the depression section evaluated 5 use cases, and the MS section evaluated 3 use cases. For each use case, there were 7 questions, which included general evaluations of practicality and benefit, plus 4 adapted from the framework of medical device design by Sharples et al [18]. Using this framework, we sought to identify the specific type of benefit offered by each use case: whether it “enabled” something new, “enhanced” existing practices, “facilitated” (made easier) existing processes, or “catalyzed” (sped up) existing processes. Respondents rated each item on a 5-point scale, ranging from 1 (strongly agree) to 5 (strongly disagree).

The “further questions” section covered clinical time, frequency of data collection, technical support requirements, usefulness or value of data, and payment and reimbursement. The “rating of concerns” section was intended to explore the extent to which different barriers to use discovered in our prior work would affect respondents’ intention to use RMT in clinical practice. Each response option in this section was more detailed and required longer to read than those in the previous sections, so we kept the number of response options low to facilitate completion (the options were “Would not prevent me using RMT with my patients”; “Would prevent use in some situations”; “Would prevent use entirely”; or “Don’t know”).

The second round survey was identical to the first, except that demographics questions were omitted, and coauthorship of the resulting paper was offered via an opt-in tick box. We chose to request full recompletion of the survey rather than only requesting completion of questions where consensus had not been reached because we were equally interested in areas of disagreement as we were in gaining consensus.

#### Follow-up Interviews

A subset of participants who indicated interest in a follow-up interview were contacted to arrange a 30-minute slot for a web-based interview using Microsoft Teams. The interviews followed a semistructured format using an interview guide instrument (Multimedia Appendix 3). The aims of the interviews were to gain further insight into HCPs’ views of the use cases for the RADAR-CNS RMT system and to understand country-specific contextual factors that might affect the implementation of RMT in each country. As such, we conducted interviews across a range of European countries and across the 3 conditions.

#### Ethics Approval

The methods were performed in accordance with relevant guidelines and regulations and were approved by the University of Nottingham Faculty of Medicine and Health Sciences Research Ethics Committee (ref: 315-0721; Multimedia Appendix 4).

#### Analysis

##### Quantitative Analysis

The quantitative data consisted of Likert-style responses from the 2 survey rounds. These were scored from 1 (strongly disagree) to 5 (strongly agree), except for the final question on barriers to RMT use, which was scored using a 3-point scale, plus an option for “do not know.” Numbers reporting “do not know” were included in the denominator of the percentage calculations.

To evaluate the consensus among respondents in each round, we used a predetermined threshold percentage of similar responses on an item [25]. We determined that consensus had been reached if 70% of the responding participants scored an item within the same grouping (agree, neutral, or disagree), where scores of 4 or 5 were grouped as “agree,” scores of 1 or 2 were grouped as “disagree,” and scores of 3 were considered neutral. This effectively recreated a 3-point scale, which is considered preferable over the analysis of 5-point scales for survey data in clinical contexts [28].

To evaluate the stability between rounds, we used the Gwet agreement coefficient, which is found to have more stable performance than kappa scores [29]. The scores were weighted to account for ordinality in the variables. The coefficients were compared against benchmarks from Altman [30].

Analyses were conducted only on responses received—no imputation was judged to be necessary to account for missing data, given that the study focused on eliciting a small number of expert opinions, with only descriptive statistics used to analyze the data.

##### Qualitative Analysis

Survey comments and interview transcripts were analyzed using template analysis [31]. An a priori theme was included in the initial template for each use case, and for each implementation topic covered in the survey. Themes were iteratively added, deleted, renamed, and reorganized to create the final template of themes. We used the final template of themes to triangulate the qualitative and quantitative data.

## Results

### Participants

A total of 23 clinicians treating patients with epilepsy, MS, or depression were recruited, with participation from all 6 European countries where RADAR-CNS is active (the United Kingdom, Germany, Spain, Denmark, the Netherlands, and Italy) and with representation from clinicians treating each of the 3 clinical conditions (Table 1). We expected some dropouts between the first and second rounds but were able to retain 74% (17/23) of the round 1 participants in round 2. A total of 9 respondents completed the interviews.

**Table 1:** Participant demographics.

		Round 1 (n=23), n (%)	Round 2 (n=17), n (%)	Interviews (n=9), n (%)
**Age group (years)**				
	30-39	6 (26)	6 (35)	1 (11)
	40-49	5 (22)	4 (24)	1 (11)
	50-59	9 (39)	5 (29)	5 (56)
	60-70	3 (13)	2 (12)	2 (22)
**Gender**				
	Woman	8 (35)	6 (35)	3 (33)
	Man	15 (65)	11 (65)	6 (66)
**Job role**				
	Consultant (medical)	16 (70)	11 (65)	8 (89)
	Health care scientist or researcher	2 (9)	2 (12)	0 (0)
	Clinical psychologist	1 (4)	1 (6)	0 (0)
	General practitioner	1 (4)	1 (6)	0 (0)
	Nurse	1 (4)	1 (6)	1 (11)
	Psychological well-being practitioner	1 (4)	0 (0)	0 (0)
	Other (unspecified)	1 (4)	1 (6)	0 (0)
**Relevant condition treated**				
	Depression	10 (43)	8 (47)	3 (33)
	Epilepsy	8 (35)	6 (35)	4 (44)
	Multiple sclerosis	3 (13)	2 (12)	1 (11)
	Multiple sclerosis, epilepsy, and depression	1 (4)	0 (0)	0 (0)
	Epilepsy and multiple sclerosis	1 (4)	1 (6)	1 (11)
**Country**				
	United Kingdom	8 (35)	5 (29)	4 (44)
	Spain	5 (22)	4 (24)	1 (11)
	Italy	4 (17)	3 (18)	0 (0)
	Germany	3 (13)	2 (12)	3 (33)
	Denmark	1 (4)	1 (6)	0 (0)
	Australia	1 (4)	1 (6)	0 (0)
	Netherlands	1 (4)	1 (6)	1 (11)

### Quantitative Results: Consensus and Stability

The research team decided that a third survey round was not required: 97.4% (114/117) of question items had a high or very high level of stability of responses between rounds 1 and 2, indicating that a third round would have had limited benefit. Multimedia Appendix 5 [30] presents the results for consensus and stability for all questions in the survey.

### Epilepsy Use Case Questions

The threshold for consensus was reached on 74% (26/35) of questions on the epilepsy use cases in the first round and 86% (30/35) of questions in the second round, demonstrating a move toward consensus. For all of these items, consensus was reached that respondents agreed or strongly agreed with the statements presented, rather than selecting “disagree,” “strongly disagree,” or “neutral.” Five of the questions moving to consensus in round 2 concerned the fifth epilepsy use case, using RMT for trend analysis, and indicated a change in views toward agreement that this use case would “enable” new possibilities, “facilitate” care (make care easier), “enhance” care, and benefit patients and clinical teams.

It is notable that in round 1, for epilepsy use cases 1 to 3, the question on “catalyzing” (speeding up) existing processes received fewer "agree" or above responses than all other statements, indicating less confidence that these use cases would speed up existing processes. The PAB identified this pattern, and their comments were fed back to the participants ahead of round 2. There was comparatively low stability for these questions between round 1 and round 2, suggesting that participants changed their minds about this question, perhaps in response to the PAB’s comments.

The point estimate for the Gwet agreement coefficient statistic fell in the “very good” strength of agreement range (0.80-1.00) for 94% (33/35) of items, indicating a very high overall stability of epilepsy responses between rounds 1 and 2.

### MS Use Case Questions

The threshold for agreement was reached on 10% (2/21) of questions on the MS use cases in the first round and 0% (0/21) of questions in the second round, demonstrating a move away from consensus. Where consensus was reached, it was a consensus that respondents “agreed” or “strongly agreed” with the statements presented rather than selecting “disagree,” “strongly disagree,” or “neutral.”

Fewer participants completed the MS use case questions in the second round (n=3) compared with the number completing epilepsy questions (n=6) and depression (n=9). This meant that even when a majority of 67% (2/3) of participants expressed an opinion, this did not cross the threshold of 70%, requiring a unanimous vote for this to occur. This explains the comparatively lower number of questions showing consensus in the MS group.

The point estimate for the Gwet agreement coefficient fell in the “very good” strength of agreement range (0.80-1.00) for 81% (17/21) of items, indicating a high overall stability of responses between rounds 1 and 2.

### Depression Use Case Questions

In the depression use cases, the threshold for agreement was reached on 83% (29/35) of questions in the first round and 89% (31/35) of questions in the second round. Where consensus was reached, it was a consensus that respondents “agreed” or “strongly agreed” with the statements presented, rather than selecting “disagree,” “strongly disagree,” or “neutral.”

The point estimate for the Gwet agreement coefficient fell in the “very good” strength of agreement range (0.80-1.00) for all 100% (35/35) of items, indicating a very high overall stability of responses between rounds 1 and 2.

### Further Questions Section

There were 19 questions on further considerations of RMT (clinical time, frequency of data collection, technical support requirements, usefulness or value of data, and payment and reimbursement), which were rated by all 17 participants who completed both rounds. Participants reached a consensus of “agree” or “strongly agree” on 42% (8/19) of questions in round 1 and maintained this level of consensus in round 2 (Multimedia Appendix 5). A smaller proportion of questions in this section reached consensus than those in the section on use cases. For 11% (2/19) of questions in this section, participants moved from no consensus to a consensus that they “disagreed” or “strongly disagreed” with the statement. These questions were that “receiving data on a patient’s condition would be an added burden” and that “mood scores need to be collected from patients at risk of mental health conditions on a daily basis.” A total of 9 question items in this range did not reach agreement in the first or second rounds. The Gwet agreement coefficient showed “very good” stability between rounds (Altman benchmark 0.80-1.00) on only 63% (12/19) of questions in this range, indicating greater changeability between rounds for these questions compared with those relating to the use cases.

### Concerns Questions

There were 7 questions on concerns about RMT, which could be rated as a serious concern (“Would prevent use entirely”), a medium concern (“Would prevent use in some situations”), or a lesser concern (“Would not prevent me using RMT with my patients”). There was no consensus for any question in this set in round 2.

The stability of responses between rounds 1 and 2 for these questions was lower than that for other parts of the survey. The change in responses was not uniform in one direction or the other, and neither was there a distinct movement toward or away from extreme responses (rating a concern as severe or lesser), indicating less certainty in relation to these questions compared with other sections of the survey.

### Qualitative Findings: Final Template and Triangulation

#### Overview

The triangulation of the results is interwoven with the overall exposition of the qualitative results below. The final template consisted of 8 themes, each with multiple subthemes (Table 2).

**Table 2:** Final template of themes and subthemes.

Theme		Subthemes
1	Comments on specific use cases	Depression (UCs^a^ 1-5, general comments) Epilepsy (UCs 1-5, general comments) Multiple sclerosis (UCs 1-3, general comments)
2	Clinical time	Implementing RMT^b^ would be time costing Implementing RMT would be time-saving Other views on RMT and clinical time
3	Value of RMT data	Disease-specific value Moving beyond the subjective Positive or negative views on value Value is related to amount of data accessible
4	Frequency, amount, and type of data collection	Collecting large amounts of data (over a year) Daily reporting and recording Desired frequency of data collection Passive data collection vs active data collection Technical support and its effect on clinical time
5	Payment and reimbursement	Funding in clinical settings to support introduction of new technologies Political drivers Requirement for extra resource Requirement to save costs or improve care
6	Country or context-specific factors relating to RMT implementation	Germany Netherlands Spain United Kingdom Setting-specific factors
7	Inevitability of change and ongoing change in health care services	Preference for at-distance care Patients use RMT and bring data to clinic Patients use RMT but don’t bring data to clinic Coronavirus pandemic as stimulus for change
8	Barriers and concerns	Comments on barriers listed in the survey Clinician time False alarms, false positives, false negatives Interoperability Patient anxiety Reducing number of appointments Other barriers not covered in the survey Requirement for further research Health care culture Legal and regulatory Patient behavior and situation

^a^UC: use case.

^b^RMT: remote measurement technology.

#### Comments on Specific Use Cases

The results on condition-specific use cases from the interviews inform the prioritization of use cases, as participants indicated which of the use cases they would find most useful and which least useful, with reasons to support these indications. Extracts from the interview transcripts for each use case are provided in Multimedia Appendix 6.

##### Epilepsy

All 5 epilepsy use cases were considered plausible, although participants stated that their utility depended on practicality and accuracy. Use cases 1, 2, and 4 (seizure alert, seizure counting, and triage support, respectively) were considered the most useful. This supported the quantitative data across both rounds.

Use case 1 (seizure alert) was considered helpful for motor seizures, which are highly associated with a sudden unexpected death in epilepsy. One participant working in Germany questioned the novelty of the solution (“we already have this for some devices” [Participant 2]), although it is understood that this is only for a patient at rest (not moving) and there is still a need for wearables that can detect motor seizures from active status. Participants indicated that an adequate level of sensitivity and specificity would be required, with 1 participant providing a detailed account of acceptable sensitivity and specificity (Multimedia Appendix 6).

Epilepsy use case 2 (seizure counting) was also considered useful, assuming appropriate levels of accuracy. Comments indicated that passive monitoring may be more accurate than patient diaries, for example, where a patient might forget to record some seizures. It was considered not to be feasible for clinicians to review data between clinic visits unless the system indicated the requirement for additional review based on particular thresholds and therefore performed some sort of triage.

Use case 3 (risk scoring) was thought to be less practical and more difficult to achieve. Interviewees thought there would be medicolegal risks and that they would not want to prevent patients from taking part in enjoyable activities where unnecessary. Use case 4 (triage support) was considered useful but less so than use cases 1 and 2. Concerns included lack of infrastructure, false positives, staffing resources, legal complications, and low availability of staff.

Use case 5 (trend analysis) was again considered potentially useful depending on evidence to support its effectiveness. Some responses to interview questions indicated that trend analysis could be one of the most useful applications of RMT in epilepsy, although quantitative findings from round 1 did not reach agreement.

##### Multiple Sclerosis

MS use case 1 (detecting silent progression) was considered useful for detecting progression early enough to slow down the condition. However, its benefits were considered to be restricted by the limited availability of medications to treat disease progression. Measuring gait was thought to be a useful mechanism for detecting silent progression (“to detect progression, the most useful would be all the tools that would be used to detect gait disorders” [Participant 3]).

There were mixed views on the usefulness of detecting depression in MS (use case 2). One interviewee indicated that the use of RMT in this way could be useful to open “a bit more conversation” with the patient (participant 19). Another interviewee stated, “it may be that detecting depression would show the development of the disease, but that would not help us so much” (participant 3). These contrasting views reflect the lack of consensus among experts in the quantitative survey results.

Use case 3 (biobanking MS data) was considered useful for future patients but not for current patients (“that it is very useful to collect this data, so I'll be interested, [...] but it will not necessarily have a direct impact to my patients” [Participant 19]), which explains the lack of consensus on the question about patient benefit. It was highlighted that there already exist biobanks for MS data and that RMT data could be added to these.

##### Depression

Interviewees indicated that use cases 1, 2, and 3 (detecting trends, reviewing treatment, and self-management, respectively) would be the most useful. Use case 1 (detecting trends) was thought to enable easier and better recording of patient-reported outcomes, which could save administrative time. Use case 2 (reviewing treatment) was considered useful if it could be implemented successfully within the treatment pathway. It was considered that use case 3 (self-management) would work well for some (but not all) patients.

It was considered that use case 4 (comorbid monitoring) might increase the rate of detecting depression (“we might encounter much more depression if we manage to do this” [Participant 12]), but this was considered the least viable use case, partly because it may not be possible to effectively treat comorbid depression if found, and partly because of concerns about confounding symptoms. These findings supported the quantitative data, where consensus was only reached on 4 of 7 questions about use case 4, compared with 6 of 7 or 7 of 7 for all other use cases. Use case 5 (carer alert) was also considered less useful, as participants thought that carers might not take on the required responsibility, being unwilling or unable to offer the right support and care.

#### General or Non–Condition-Specific Questions

##### Clinical Time

The responses showed that implementing RMT could be overall time saving if it reduced admissions, although the potential for RMT to identify otherwise unidentified symptoms may in fact require more clinical time to evaluate. RMT may reduce emergency department burden, where conditions are better managed. Comments suggested that time saving would depend on high accuracy. Several participants described practical ways in which RMT could be used to save time, eg, the use of thresholding, and having a patient manager specifically trained to manage RMT data. Some also suggested that the value of RMT may be in having a more detailed picture to improve care rather than saving time. Interviewees stated that having good quality, easily available technical support could save time for clinicians and encourage the continued use of RMT, although some were concerned about the cost of technical support.

##### Value of RMT Data

Interviewees suggested that RMT data would be useful in conditions outside the 3 covered in RADAR-CNS, eg, in the monitoring of bipolar disorder:

I wonder whether you also consider bipolar disorder if you talk about depression, I think you have to. Even if patients come from the unipolar depression side, they still might switch into mania.

Participant 11

To some extent, the value of the RMT data was correlated with the amount of data that could feasibly be collected. It was considered that “the more data you have, the less uncertainty there is” (participant 4) and that the data could give otherwise unavailable insights into patient condition:

Having the RMT background information in terms of their activities throughout the week, I think it will probably give us a little bit more information in terms of how they’ve been during the week rather than, you know, on that day, this is what they reported.

Participant 5

This linked with a wider subtheme on “moving beyond the subjective.” Interviewees stated that RMT could provide objective data that would otherwise be represented only by subjective patient self-reports. It was also considered that RMT would enable clinicians to determine how much the patient’s condition affected their everyday lives even when the patient said they were “fine.”

##### Frequency, Amount, and Type of Data Collection

The required frequency of data collection would depend on the stage of the disease and treatment phase. In relation to mood, participants’ desired frequency of mood report data ranged from daily to once fortnightly with various suggestions in between.

There was a suggestion that passive data collection was more valuable than active data collection, as compliance with active measures was expected to be low and because active measures can have the undesirable effect of inducing negative mood states:

From another trial, assisted active monitoring can even induce bad mood states because people then tend to ruminate, tend to think about the situation more than they probably should. So it should stress passive monitoring.

Participant 11

##### Payment and Reimbursement

Many participants expressed that the introduction of RMT in these use cases would require a large amount of extra resources, for the cost of devices, for staff who would monitor patient data, and for staff members who would help patients set up the technology. Interviewees also expressed that the essential requirement for the introduction of RMT would be that it could demonstrably save costs or provide strong evidence of an improvement in patient care:

Whatever you implement must not increase your workload, because otherwise especially doctors in their own practice won’t use it because they don’t get any extra money for that.

So they must see a time saving benefit or a real quality improvement for patient care. That is what they expect, and here it's really important to stress that it's not putting extra work on doctors, but makes life easier actually.

Participant 11

There were mixed views on whether reducing the number of patient appointments, as a result of monitoring their condition remotely, would be useful. This reflected the quantitative survey results, where 9 of 17 reported that their service would lose money if appointments were reduced and 7 of 17 reported that their service would not. Payment regulations for a clinician treating epilepsy in Germany meant that a reduction in the number of appointments would cause a reduction in income for his service. He stated that a political change would be required to incentivize the use of new wearable solutions in epilepsy. Conversely, a clinician treating epilepsy in the United Kingdom stated that where appointments were saved for 1 patient, these would be filled by another, as the demand for the service was so great: “There’s too much demand, so don’t worry about dropping income because of dropping demands” (participant 4).

#### Country- or Context-Specific Factors Relating to RMT Implementation

##### Germany

Interviewees in Germany gave mixed reports on the potential for the reimbursement of RMT. One interviewee said that some wearable devices were already provided to patients with epilepsy, paid for by the health service, providing evidence of a precedent for the funding of RMT. They added that RMT may have limited cost-saving benefits because of the requirement for additional staff. Another interviewee said that the organization of remuneration for health care in Germany is old fashioned and limits the ability to introduce new technologies. Another said that where doctors run their own clinics, they are free to use any technologies they see fit as long as they can convince their budget manager of the benefit the new technology will offer. This interviewee also mentioned the German law introduced in recent years to incentivize the introduction of digital health technologies and described the requirement for these technologies to provide strong supporting evidence:

They need to demonstrate, well, evidence for helpfulness. It’s not the level of a randomized control trial, but they need to have data that the app would be instrumental in reducing health care burden, and then the provider gets reimbursed. So it’s like prescribing a medication or something like that.

Participant 11

##### The Netherlands

The single interviewee from the Netherlands explained that there is hope and enthusiasm that RMT may offer patient and health care service benefits in depression in the Netherlands, but that there is as yet little implementation. They contrasted the National Health Service (NHS) in the Netherlands with the situation in Germany, where it was perceived that individual German hospitals needed to attract patients and that RMT may offer a competitive advantage, whereas the Dutch NHS did not need to do so.

##### Spain

The interviewee treating patients with MS in Spain suggested that regulatory factors might complicate the introduction of RMT in Spain and that there was little money in the Spanish health service to introduce RMT. However, they mentioned that because of the COVID-19 pandemic, many patients with MS whose condition is stable now have remote visits via videoconferencing as a matter of course, which have laid the cultural groundwork for a change in patient monitoring and management.

##### The United Kingdom

Interviewees distinguished the United Kingdom from other countries by highlighting how health care practitioners treating MS in other countries may be paid per visit, and it is thus in their interest to have patients attend clinics. However, in the United Kingdom, no such pay-per-appointment system is in place. Therefore, UK practitioners may be more keen than those in other countries to make the best use of RMT data to cancel appointments where unnecessary.

It was also highlighted that insurer-based health systems compete for patients, but the UK NHS does not, so there would be less motivation to introduce RMT as a competitive advantage in the NHS.

Although interviewees pointed out that there is a political push for increased implementation of digital solutions in the NHS, 1 interviewee suggested it would be politically unpopular for the NHS to offer consumer-grade electronic goods for health-related purposes free on the NHS:

Say with an Apple Watch retail price, probably three, 400 pounds, I don’t know. You could see the social envy creeping up and saying, oh I’m not paying for epilepsy patients to get an Apple Watch which I can’t afford myself so consumer electronics is one thing.

Participant 9

Similar to other countries, UK interviewees stated that funding for new medical technologies is focused on research trials rather than on implementation. They stated that implementation is assumed and is not highly regarded in terms of researcher or practitioner prestige.

##### Setting-Specific Factors

In relation to clinical alert-based systems (ie, those where crossing a threshold in patient RMT data may trigger an alert to a medical team), interviewees stated that these would have easier applications in acute hospital settings rather than in community-based settings. However, there was concern that larger centers or hospitals would be likely to see greater adoption of remote technologies because they have more funds available to cover excess costs and that this would contribute to inequality:

You end up having three hospitals that they are already providing a good care to provide a bit better care. So if anything, the inequality of care will widen.

Participant 19

#### Inevitability of Change in Health Services

Interviewees commented that changes were inevitable in health care services and that pathways and procedures are often evolving. In relation to RMT, some interviewees indicated that they were aware of patients already using wearables to monitor health, with some of these sharing data with clinicians (and some not). The coronavirus pandemic was discussed as a stimulus for lasting change that may lay the groundwork for the future implementation of different types of RMT. Respondents reported that some patients were fearful of face-to-face contact with health care practitioners in light of the threat of COVID-19.

### Barriers and Concerns

#### Barriers Covered in the Survey

There were mixed views on whether false positives and false negatives from RMT would be problematic, reflecting the lack of consensus in this area in the quantitative results. One interviewee stated that false positives or negatives would not undermine the usefulness of RMT, as such results can be expected from any measure, whether digital or analog. Interviewees also mentioned interoperability and patient anxiety (covered in the survey), where if RMT made patients more anxious, this could drive the increased use of clinical resources, which could be problematic. Another interviewee mentioned that carers may be made more anxious by the introduction of RMT:

Sometimes parents can sometimes focus too much on stuff that’s not relevant, and then that gets in the way of them focusing on the more important things.

Participant 4

#### Barriers Not Covered in the Survey

There was concern among interviewees that patients may not adhere well to monitoring regimes; that devices would be lost, stolen, or sold; or that patients may not have suitable internet or mobile data to enable the use of RMT. Other worries were that patients may buy cheap, less accurate, and unregulated devices if the appropriate devices are not provided for free and that the implementation of RMT would only be successful if patients believed that it would work, requiring adequate patient education.

Health care culture was identified as an important barrier. It was suggested that HCPs were often unaware of what technologies are readily available to support their patients. Interviewees stated that, in the United Kingdom, it is very difficult for an HCP to persuade more senior staff members of the necessity for any particular kind of technology that they were aware of:

They would say, oh, you gotta do a business case if you want to introduce new technology. The business case is quite difficult to do. There’s very little admin support for it, unless it’s a very high priority of the trust.

Participant 4

Legal and regulatory systems were also highlighted, with interviewees suggesting that these are not currently set up for technologies that are recurrently updated, eg, algorithms that update themselves. Data protection and ownership were also mentioned as key issues worthy of consideration when implementing new technologies.

Further research is necessary to determine the accuracy and reliability of off-the-shelf consumer technologies used within the system. CIs for their precision would be required to make use of these parameters successfully. The participants recommended trials of the specific use cases of the technologies under development to establish cost-effectiveness.

## Discussion

### Overview

The purpose of this mixed methods, sequential explanatory Delphi study was to prioritize among use cases for RMT in central nervous system disorders, which had been cocreated with clinicians and patients within the RADAR-CNS consortium. The results from the study have identified those likely to be of the most practical use and clinical benefit. The study has also contributed knowledge on country- and context-specific factors affecting implementation and revealed areas of consensus and disagreement among HCPs on practical aspects of RMT implementation.

### Principal Findings

#### Epilepsy

Priority use cases for RADAR-CNS RMT in epilepsy from this study are: seizure alert, seizure counting, triage support, and trend analysis. All 5 use cases for epilepsy were considered “beneficial to patients”; however, use case 3 (risk scoring) was considered less likely to facilitate or catalyze care or be beneficial to clinical teams. Participants suggested that risk scoring would bring medicolegal risks and would be less practical and more difficult to achieve than other use cases.

Although other authors have commented on the potential of technology in these areas [32-34], our study has validated these as the most useful applications of RMT with clinicians treating epilepsy across Europe. Participants in the study were concerned about the possible medicolegal consequences of using devices to estimate the risk of seizures in epilepsy, which might be expected, given that prior work has highlighted the medicolegal responsibilities of clinicians treating patients with epilepsy in relation to driving and employment in the teaching profession [35].

#### Multiple Sclerosis

There was little consensus on the benefits of the use cases in MS. Despite repeated efforts to avoid dropout, the results in round 2 were only obtained from 3 participants, limiting the robustness of these results. There was greater consensus among the 6 participants completing round 1, particularly for use case 1 (detecting silent progression) and use case 2 (detecting depression in MS). The qualitative findings showed some dependencies, eg, that the system could hold value for the detection of silent progression of the disease if relevant treatments are available, whose delivery could be optimized by applying them at specific times relevant to the timely detection of changes by the system.

On reviewing our findings, patients commented that cognition is an important aspect of MS to measure the silent progression of the disease. They also mentioned that compliance with monitoring programs may be greater where they experienced a decline or instability in their condition. Related work has found that clinicians have concerns about collecting data with little clinical relevance [3,36], and our work illustrates this in the specific case of MS, where HCPs were skeptical about the benefits of detecting silent progression if no treatments were available to address it.

The UK Biobank now includes data on sleep and physical activity from wearable devices, and researchers have begun to analyze these data for various purposes [37]. Here, we provide evidence that some clinicians support such storage of data from patients with MS, although some are unclear about the benefits to the donating patient. Issues around the security and privacy of patient wearable data in biobanks were mentioned by the interviewees. The World Medical Association has adopted a declaration on ethical considerations regarding health databases and biobanks [38], and such issues will need to be duly considered for any future storage and sharing of wearable and smartphone sensor data in biobanks.

#### Depression

The use cases to be prioritized for RMT in depression include detecting trends, reviewing treatment, and self-management. Participants agreed that there would be benefits for all 5 of the depression use cases, although there was less consensus for use case 4 (comorbid monitoring), where qualitative results showed that participants thought it would be difficult to distinguish between symptoms of depression and symptoms of comorbid physical illnesses. Use case 5 (carer alert) was criticized in the interviews, as informal carers may not have the requisite skills or knowledge to adequately support patients with depression. Use cases 1 to 3 were seen as useful provided evidence could be generated to support their effectiveness.

Self-management was one of the most favored use cases for RMT in depression. There is some evidence supporting the effectiveness of smartphone apps for depression self-management [39], although qualitative evidence shows that users may download apps for short-term, inquisitive trials and may not adhere for longer term use [40]. Further work is required to establish what factors affect adherence to depression self-management apps and how the RADAR-CNS RMT system can be presented to patients to encourage continued use.

Other use cases supported by participants for depression were use case 1, detecting trends and use case 2, reviewing treatment, including monitoring of treatment response and side effects. Existing methods of detecting trends and monitoring treatment response rely predominantly on pen-and-paper mood diaries and outcome measures, such as the Patient Health Questionnaire-9. However, many such outcome measures have been converted to digital versions [41-43], and electronic mood diaries are also becoming available as smartphone or web applications [44,45], with some efforts to automatically detect symptoms and analyze trends from these user-entered data [2]. The multimodal, passive, and active combinations that could be offered using the RADAR-CNS RMT system are less commonly available, although some research has begun in this area [46]. This type of approach likely requires a higher level of regulatory approval than electronic mood diaries [47].

#### Further Questions

There was lower consensus and stability on these questions than those relating to the use cases, suggesting more differences of opinion and less fixed views on these issues. However, our findings clarify some points: It was expected that the implementation of RMT would require greater amounts of staff time and financial resources than the status quo. Evidence of cost-effectiveness was considered imperative. The RMT data were considered valuable for reducing uncertainty and moving beyond subjective measures. It has been suggested that RMT could offer benefits under conditions other than the 3 under consideration in RADAR-CNS, eg, bipolar disorder. There were mixed views on how frequently the data should be collected. Passive data were considered more useful than actively collected data because they required less input from the patient, who may forget to complete questionnaires and because passive data were considered to be less subjective.

Country-specific comments highlighted the difference between countries with NHSs (the United Kingdom, the Netherlands, and Spain) compared with countries with insurer-based health care systems (Germany). It was mentioned that RMT could be used to persuade customers to join 1 particular insurer or health service over another. Differences were also highlighted where countries may have a pay-per-patient-visit setup, wherein the use of RMT to check patients are stable and therefore reduce appointments would be financially problematic. The interviews covered only a limited range of countries, and there may be other barriers or facilitators to using RMT in health care systems in other countries. Barriers were raised regarding inequality between settings, patient behavior, health care culture, legal or regulatory issues, and use of off-the-shelf technologies.

### Limitations

We would caution against overinterpretation of the consensus scores for MS, where only 3 participants responded in the second round. Unfortunately, we could not recruit more experts in MS during the time available for the study, despite extending the recruitment window and using multiple recruitment methods. This is a shortcoming of many studies seeking the views of MS clinicians, as there are few medics specializing in this condition. However, the combination of item ratings and interview findings relating to MS provides useful insights into how RMT could be used for patient benefits in this condition.

In addition, the aim of this study was to explore the applicability of the RADAR-CNS RMT system in 3 central nervous system disorders, limiting its relevance to other conditions or monitoring platforms. However, the methodology set out here will likely be of interest to others seeking approaches to evaluate the application of novel systems in health care, and the findings will be of interest to those developing a variety of digital interventions for the specific conditions discussed.

### Interpretation and Implications for Research and Practice

Our work highlights the potential value of the implementation of RMT for 3 central nervous system disorders, including which applications of RMT would enable something new, enhance existing care, speed up existing processes, or facilitate or make easier the care of patients [14,18]. These results indicate that clinicians would consider RMT patient data to have sufficient value that it would be worth a financial outlay to implement RMT in clinical practice. However, health economic evaluation is required to determine the cost-effectiveness of applying RMT in each of these conditions [48], and the choice to implement is likely to be determined by whether cost-effectiveness is judged to meet local criteria that can vary by country [49].

The findings from this study and our prior work provide an indication of where costs may be incurred if RMT is implemented in a health care service [17]. The costs are likely to include introducing staff roles to manage patient data and provide technical support. Technical support staff could assist patients in setting up and maintaining RMT devices and support clinicians in making the best use of patient RMT data. The extra time for clinical staff to review patient data would be cost incurring, where fewer patients can be seen, although if this results in improvements in care and thereby patient condition, there may be an overall improvement in efficiency. The devices themselves and their maintenance also incur a cost for a health care service wishing to implement them. Although many of the technical devices incorporated within the RADAR-CNS system are consumer-grade technologies that may be owned by patients, our findings suggest that there is a need to provide each patient with devices meeting specific standards of accuracy, adding to costs.

Participants largely suggested that decisions about the implementation of new technologies were top-down and that commissioners and health service leaders would need to be convinced of the benefits of RMT for it to be implemented. In the United Kingdom, commissioners are often involved in redesigning services to incorporate new and beneficial technologies or products [50]. The exception to this was Germany, where doctors who run their own services at a local level are able to work with their budget holders to decide on the implementation of particular technologies. However, it is expected that these technologies should be demonstrably both clinically effective and cost-effective. In the United Kingdom, the National Institute of Health and Care Excellence provides guidance on the evidence required for approval of digital technologies [51]. Similarly, in the European Union, the European Medicines Agency works with groups to develop novel health care technologies to provide scientific advice [52]. Close working with these organizations would facilitate the further development and evaluation of the RADAR-CNS RMT system.

### Conclusions

RMT offers new possibilities for the assessment of epilepsy, MS, and depression by enabling new ways of caring for patients, enhancing existing processes, facilitating care, and, in some areas, catalyzing or speeding up existing processes. Our study shows promise for the use of wearable technologies such as Fitbits, wrist-worn epilepsy seizure detection devices, and other wearable accelerometers, together with smartphones, in remote measurement and assessment systems. Priority use cases for the further development and evaluation of RMT in epilepsy according to this study are: more accurate seizure records, automatically analyzing trends, improving triage through review of RMT data, and alerting patients and carers to imminent seizures. In depression, priority use cases are using RMT to detect trends or changes in the condition, monitoring treatment response and using data to inform treatment decisions, and self-management through monitoring and behavioral nudges. Some clinicians recognize the benefits of RMT in the management of MS by enabling the detection of the silent progression of the disease, detecting depression, and enabling the donation of data to biobanks, although clear priorities among these cannot be distinguished from our results.

The implementation of RMT will have different implications in different health service models. Cost-effectiveness studies will be required to understand the economic value of implementing RMT in clinical practice in different regions. Future work could also usefully explore the potential of RMT in other clinical conditions, as well as seeking to understand factors affecting adherence to remote measurement regimes in real-world conditions. Clinicians participating in this study considered passive data to be more reliable than active data, and further work is required to understand whether digital biomarkers based on passive remote measurement data can be used as proxies or replacements for existing measures in these and other clinical conditions. Overall, the Delphi method has been useful for prioritizing use cases and deriving insights into the practical application of RMT in clinical practice.

## Appendix

Multimedia Appendix 1 Illustrations of final use cases.

Multimedia Appendix 2 Delphi study survey instrument.

Multimedia Appendix 3 Interview guide.

Multimedia Appendix 4 Research Ethics Committee Letter of Approval for Delphi Study.

Multimedia Appendix 5 Results of consensus and stability analyses.

Multimedia Appendix 6 Illustrative extracts from interview transcripts relating to each use case.

## Abbreviations

MS: multiple sclerosis
NHS: National Health Service
PAB: Patient Advisory Board
RADAR-CNS: Remote Assessment of Disease and Relapse–Central Nervous System
RMT: remote measurement technology

## Data Availability

The data from the study may be requested from the authors and will be made available on reasonable request, on approval by all members of the Remote Assessment of Disease and Relapse—Central Nervous System consortium, in accordance with the contractual agreements in place between institutions. A bilateral agreement must be entered to determine the terms and conditions of data sharing.
