# Supplementary figures and images for: Clinical Perspectives on Using Remote Measurement Technology in Assessing Epilepsy, Multiple Sclerosis, and Depression: Delphi Study
Source: JMIR Neurotechnol. 2023 Apr 25;2:e41439. doi: 10.2196/41439 (PMC12671310; doi:10.2196/41439)

# Multimedia Appendix 1: Final Use Case Diagrams

**Depression**


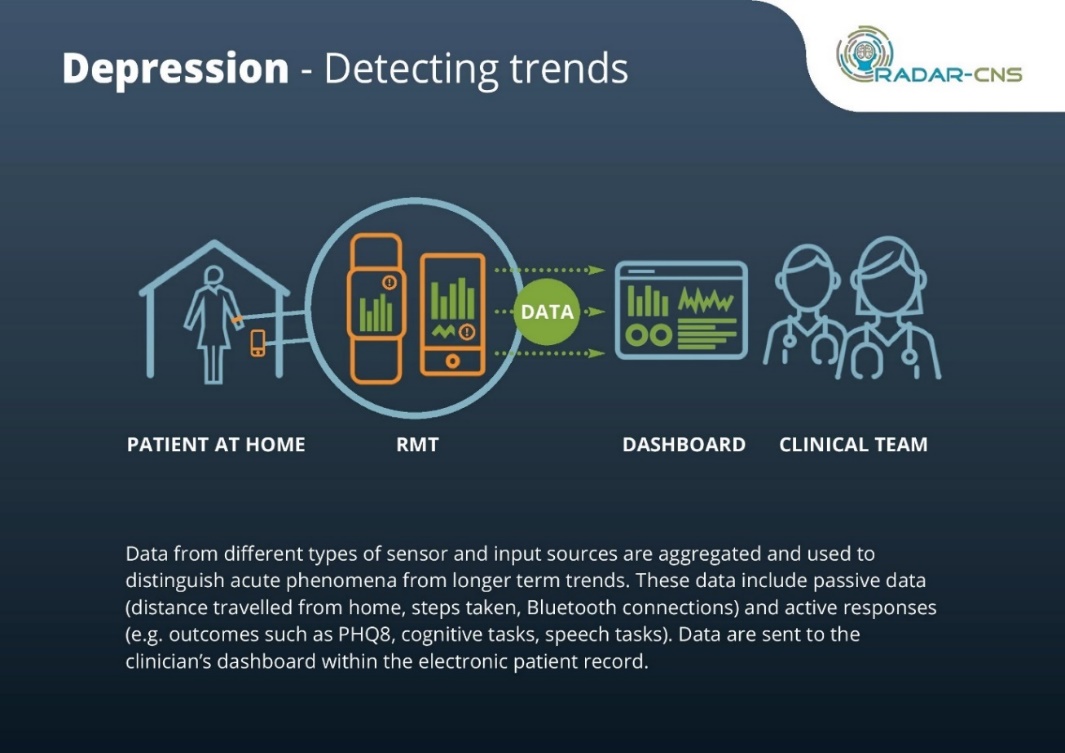

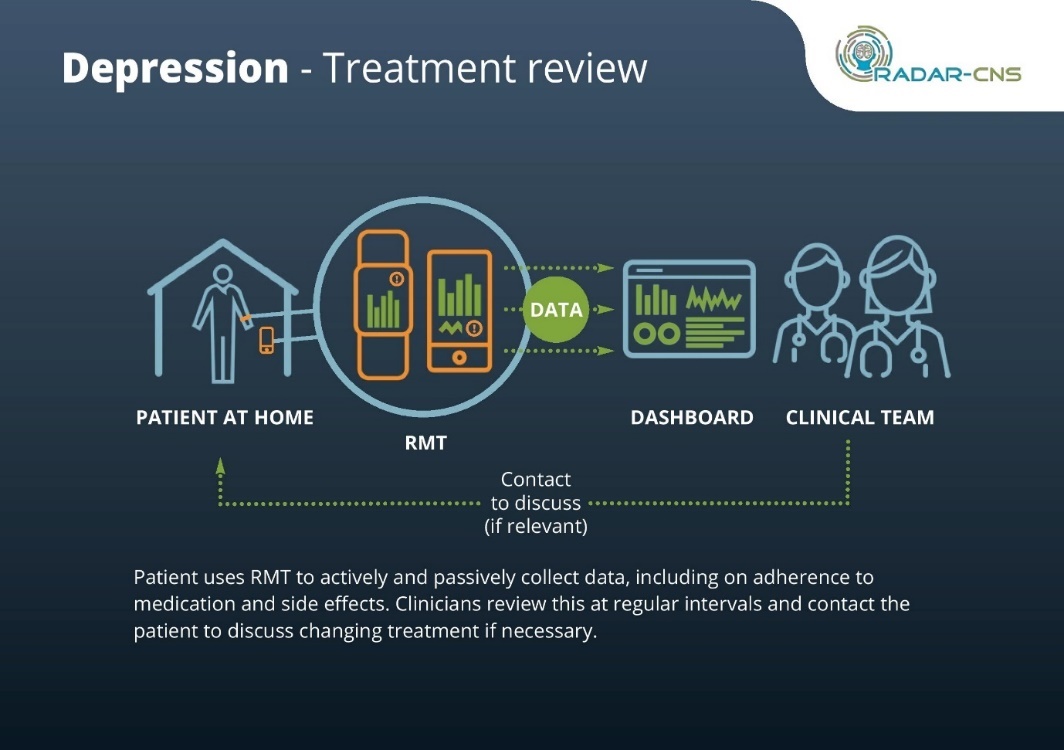

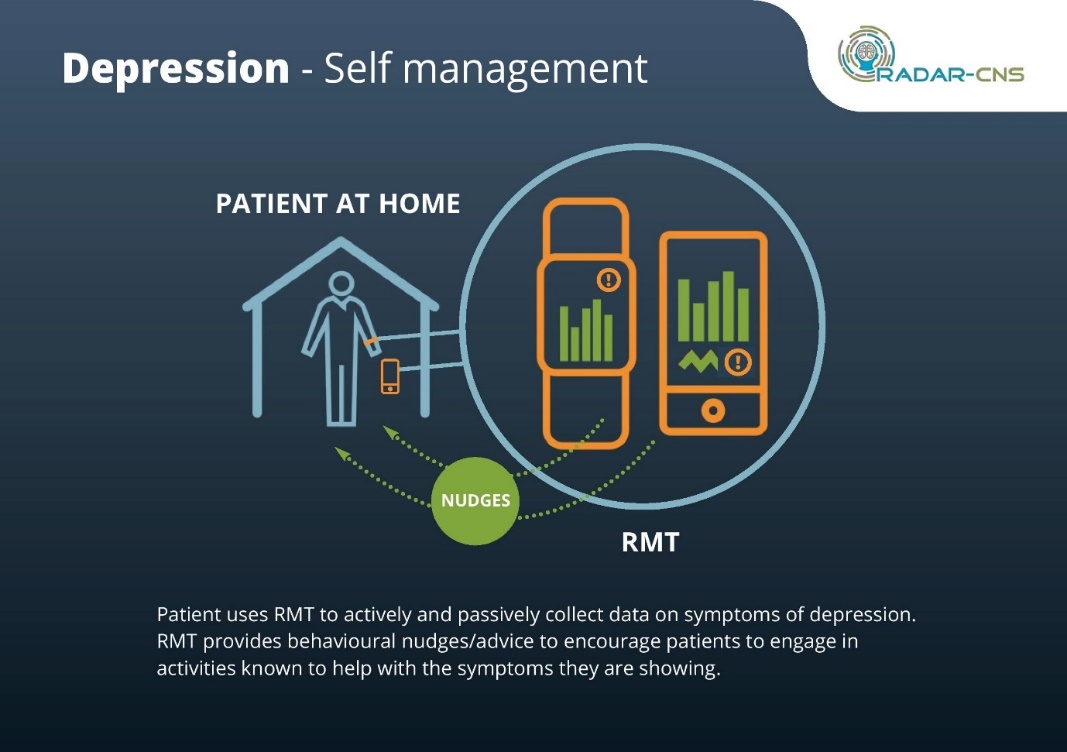


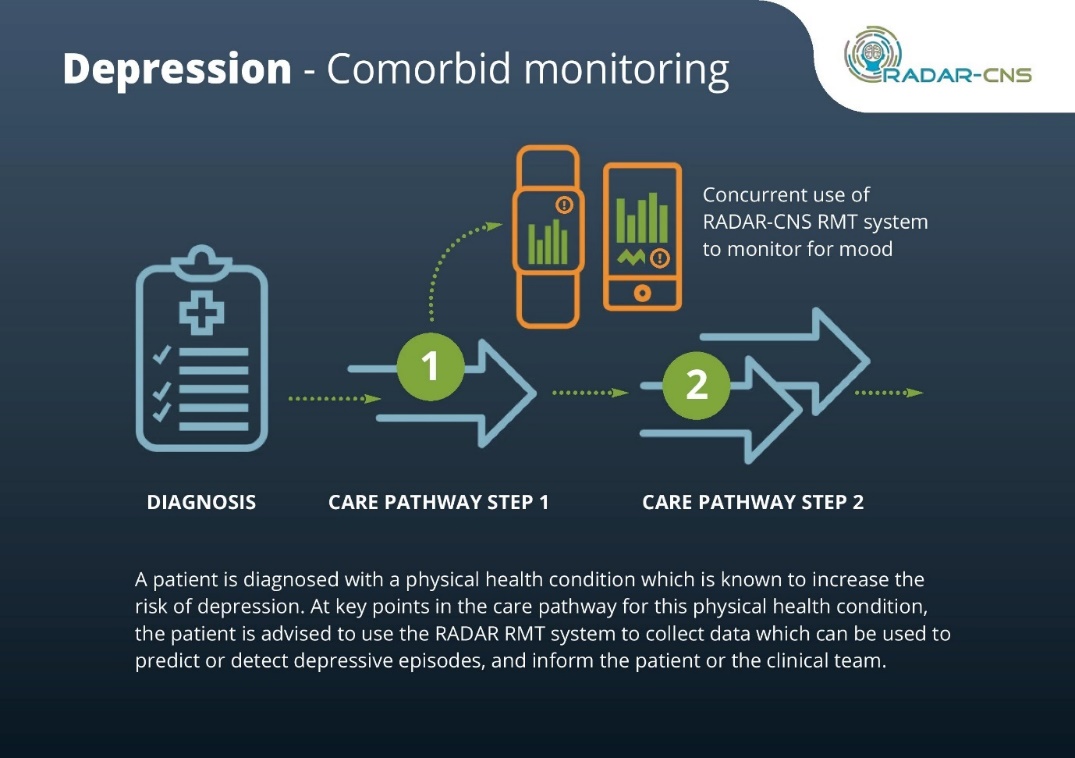

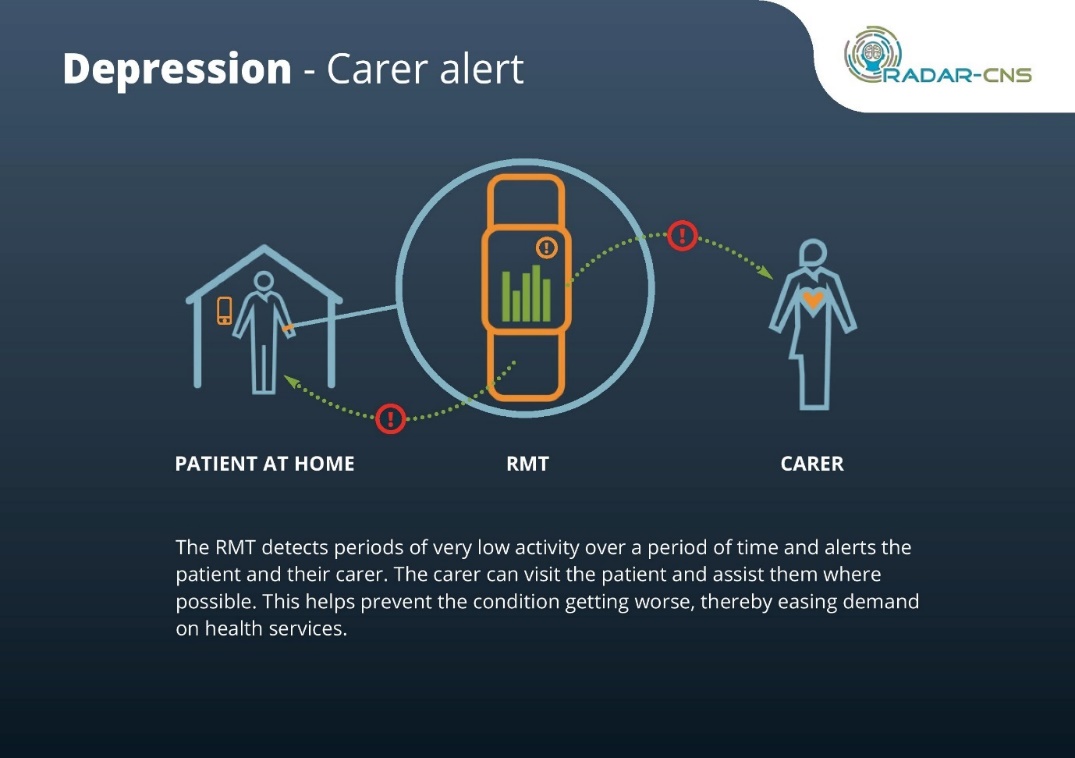


**Epilepsy**


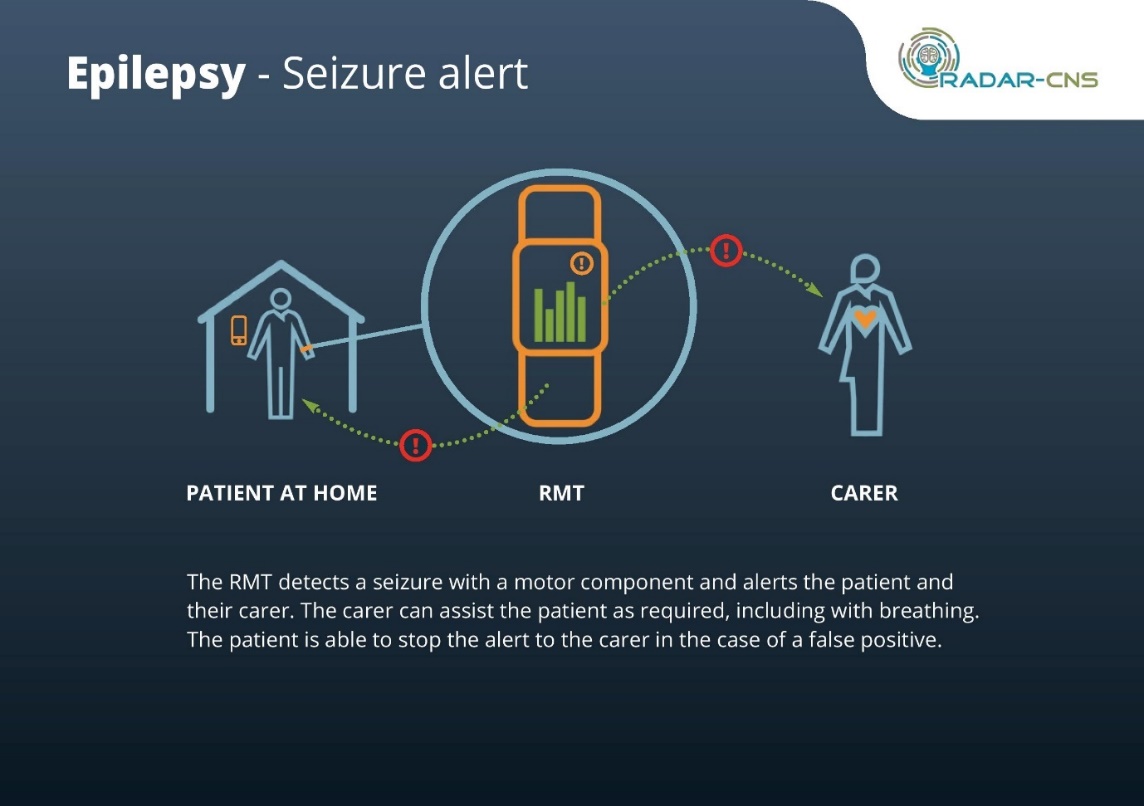

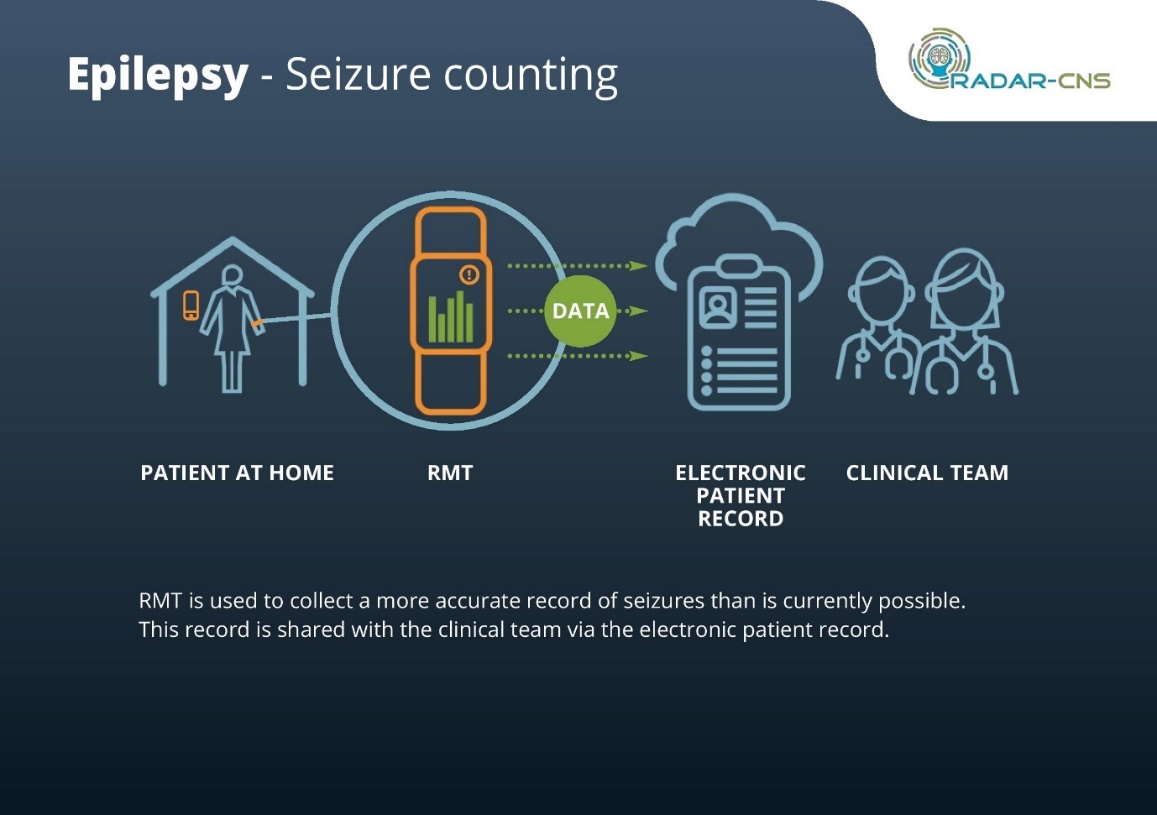

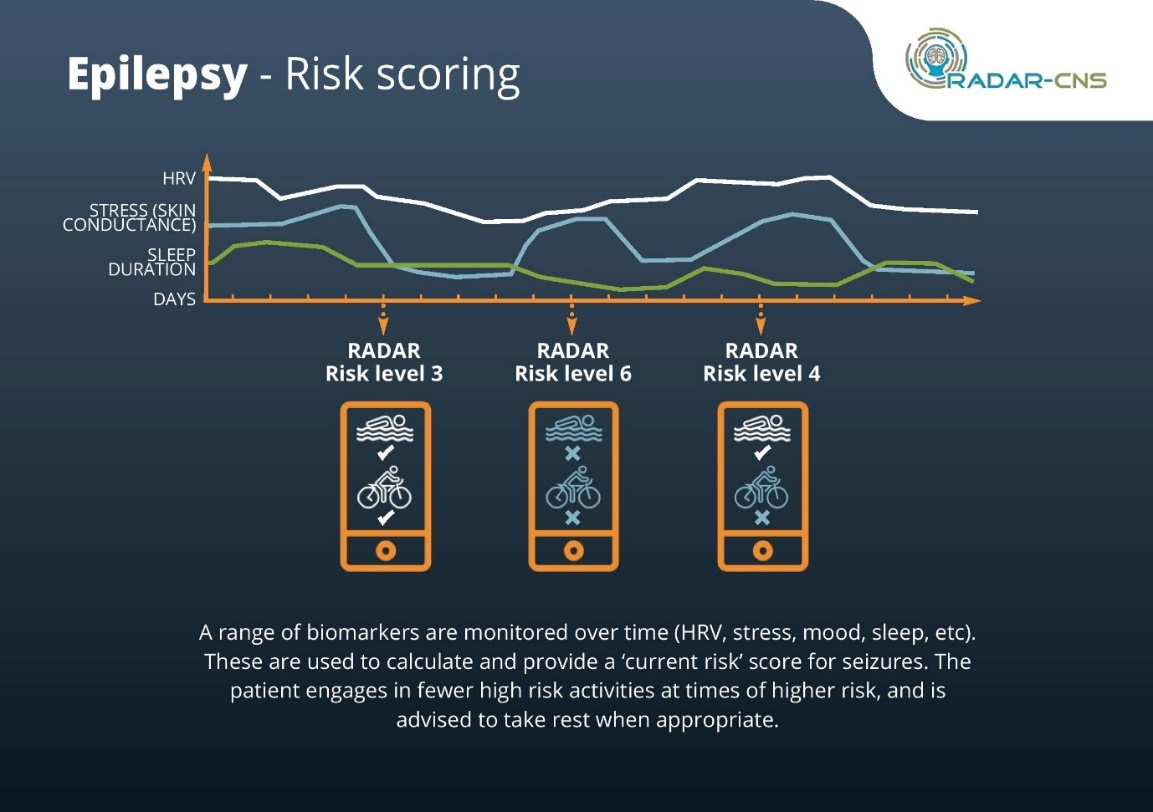

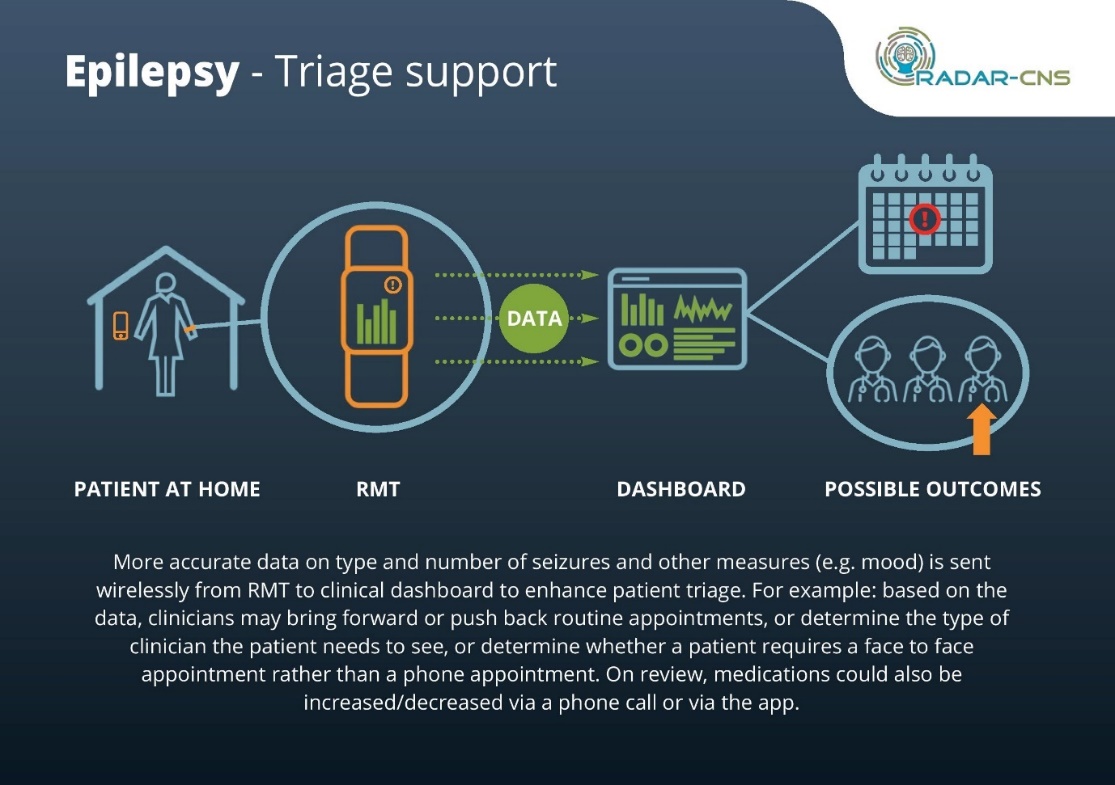

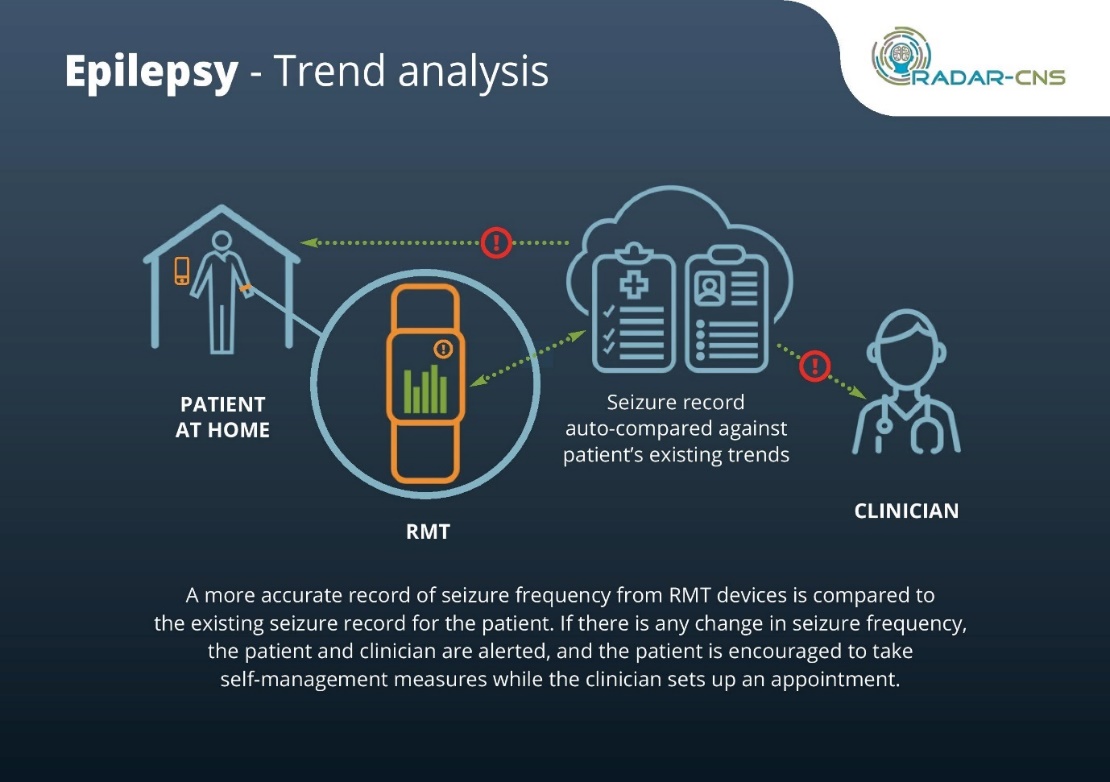


**Multiple Sclerosis**


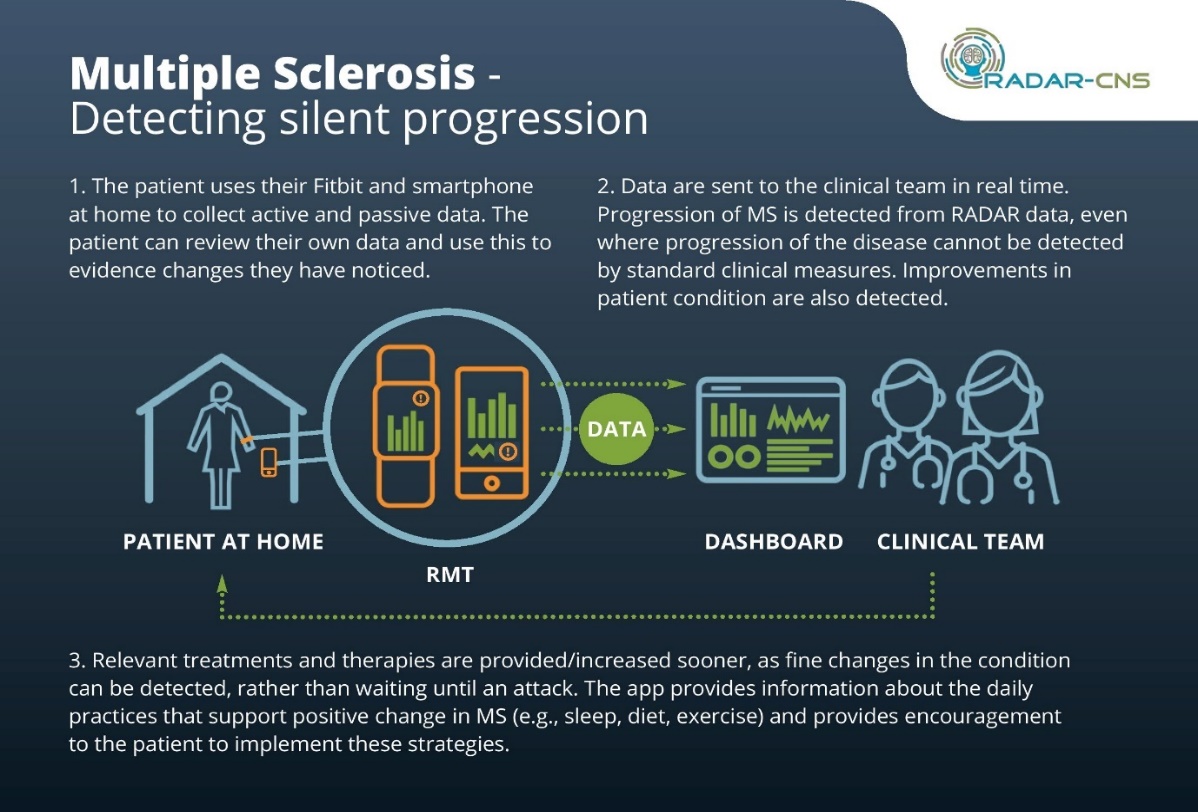

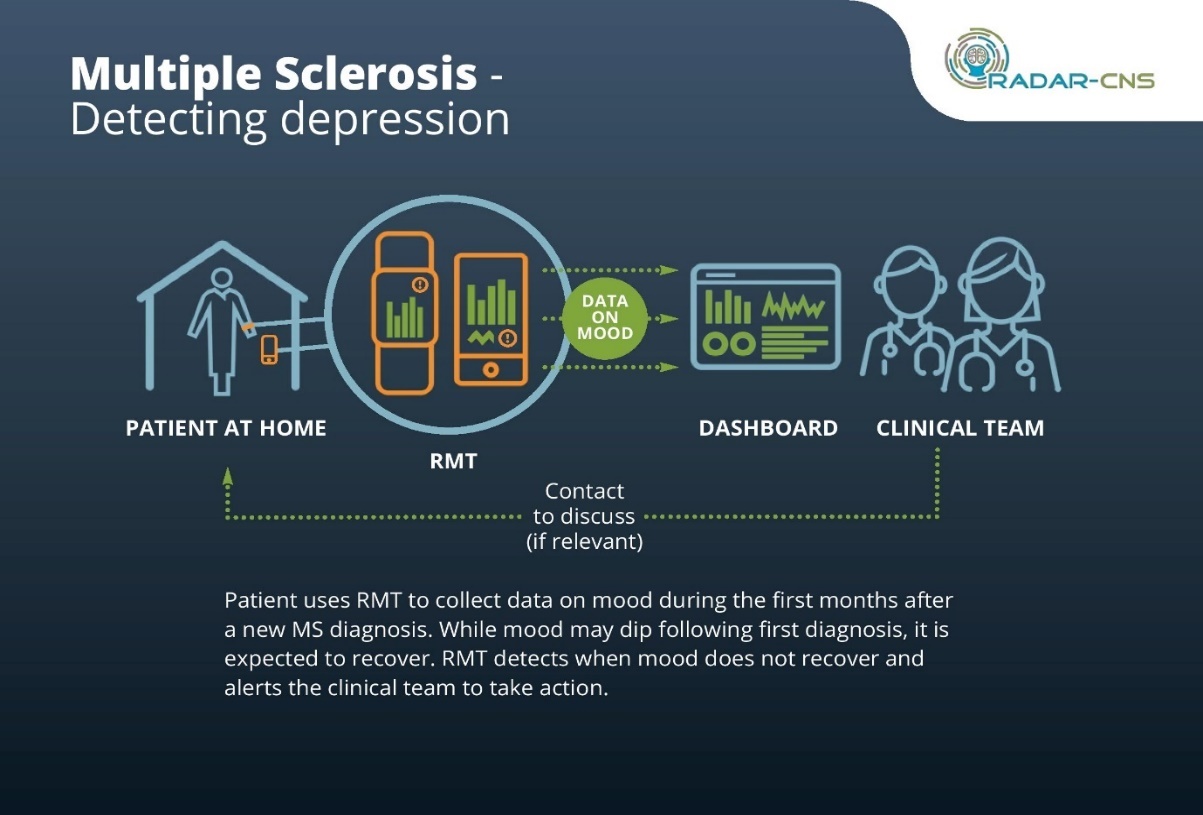

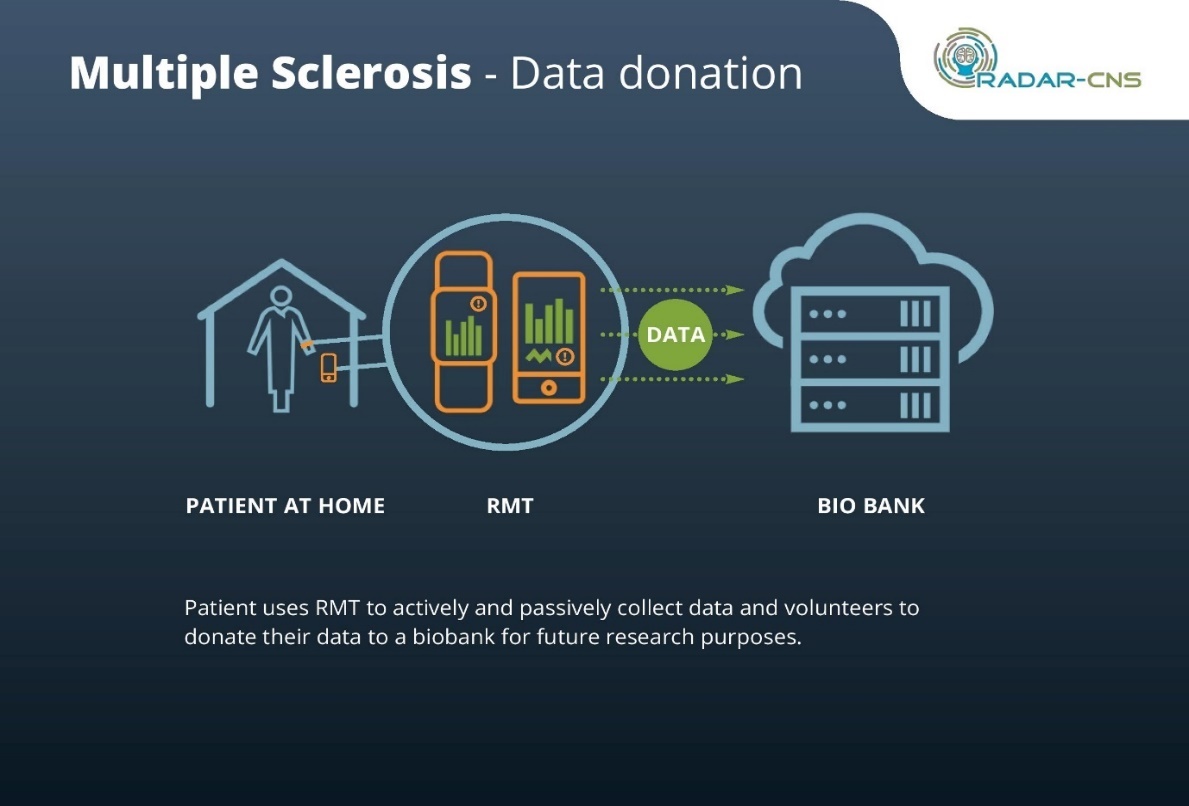

Supplement: Multimedia Appendix 1 [file neuro_v2i1e41439_app1.docx]

# Multimedia Appendix 4: Research Ethics Committee Letter of Approval for Delphi Study


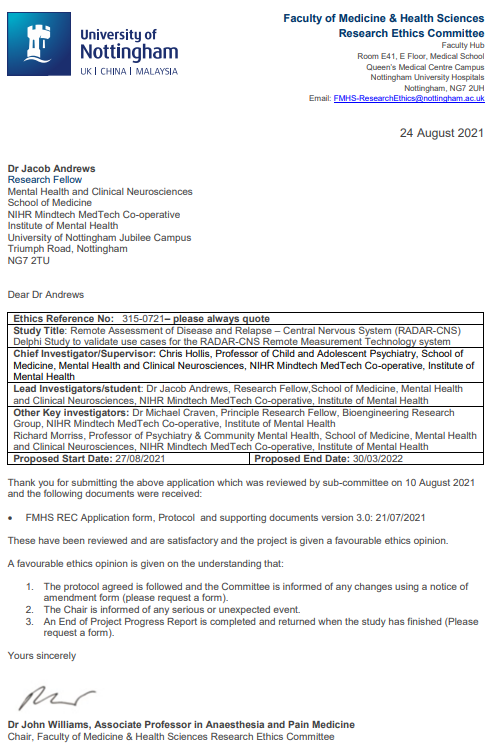

Supplement: Multimedia Appendix 4 [file neuro_v2i1e41439_app4.docx]
