# Supplementary material for: Clinical Perspectives on Using Remote Measurement Technology in Assessing Epilepsy, Multiple Sclerosis, and Depression: Delphi Study
Source: JMIR Neurotechnol. 2023 Apr 25;2:e41439. doi: 10.2196/41439 (PMC12671310; doi:10.2196/41439)
Supplement: Multimedia Appendix 2 [file neuro_v2i1e41439_app2.docx]

# Multimedia Appendix 2: Delphi study survey

**RADAR-CNS Remote Monitoring Technology Delphi Survey**

**Part 1 - Demographics**

Thank you very much for completing the consent form and agreeing to take part in this study.

This study is a Delphi study consisting of multiple rounds. In this first round, we would like you to respond to the following questions on face value. In the second round (late August), we will provide you with the views of other survey completers and invite you to reconsider your responses. This is a commonly used method for seeking consensus.

We'd like to start by collecting some demographic information.

1. Which of the following conditions do you treat in your clinical practice? Please tick all that apply:
   1. MS
   2. Epilepsy
   3. Depression
2. What is your age? (optional)

Reason: We would like to explore whether there are any differences in answers to the survey based on age.

- 1. [free text response]

1. What gender do you identify as? (optional)

Reason: We would like to explore whether there are any differences in answers to the survey based on gender.

- 1. -----

1. What of these most closely describes your job role?
   1. General Practitioner
   2. Consultant
   3. Junior doctor
   4. Nurse
   5. Allied Health Professional
   6. Medical student
   7. Healthcare scientist
   8. Clinical psychologist
   9. Psychological wellbeing practitioner
   10. Wider healthcare team
   11. Other
2. What is your clinical setting?
   1. Primary care/general practice
   2. Secondary care, hospital setting
   3. Secondary care, community setting
   4. Specialist tertiary care
   5. Community care
   6. Other
3. Which country do you work in?
   1. UK
   2. Spain
   3. Italy
   4. Denmark
   5. Holland/the Netherlands
   6. Germany
   7. Other [free text response]

**Part 2 – Clinical use cases for the RADAR-CNS RMT system**

We have developed diagrams that illustrate how remote measurement technologies (RMT), consisting of wearable wrist straps and smartphone apps, could be used in clinical practice. We invite you to examine these 'use cases' for each condition you have experience treating, and respond to the questions that follow.

***[Note:*** In the online survey, each use case image in Appendix 1 was presented in turn, followed by the same 7 questions below for participants to rate for each. The image below is only the first one, as an example. Branching was used to ensure participants were only shown questions for conditions that they treat.***]***


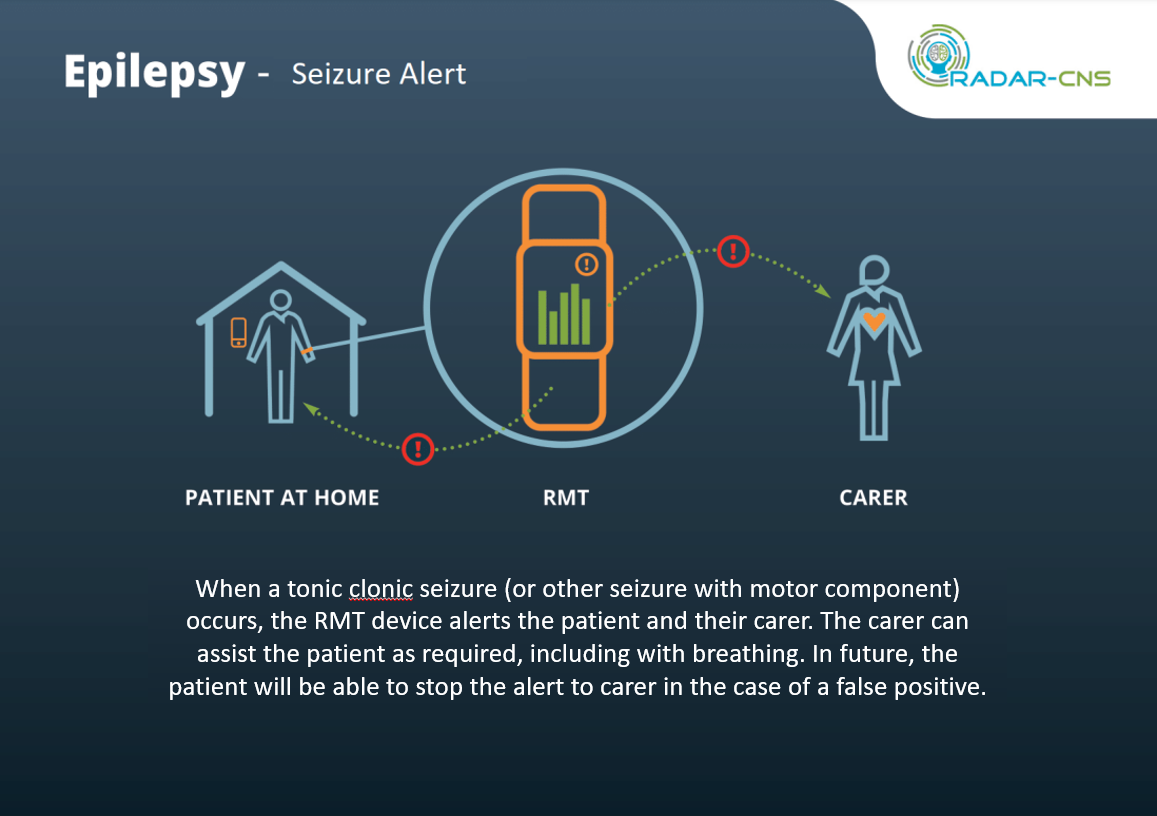


Please indicate whether you **strongly agree, agree, neither agree nor disagree, disagree or strongly disagree** with each of the following statements in relation to the use case illustrated above.

**I think it would be practically possible to employ RMT for this use in the care of my patients.**

strongly agree / agree / neither agree nor disagree / disagree / strongly disagree

**I think this use of RMT would enable (make possible) something which isn’t currently possible.**

strongly agree / agree / neither agree nor disagree / disagree / strongly disagree

**I think this use of RMT would facilitate (make easier) the care of my patients.**

strongly agree / agree / neither agree nor disagree / disagree / strongly disagree

**I think this use of RMT would enhance (add to) the care I give to my patients.**

strongly agree / agree / neither agree nor disagree / disagree / strongly disagree

**I think this use of RMT would catalyse (speed up) processes in the care of my patients.**

strongly agree / agree / neither agree nor disagree / disagree / strongly disagree

**Overall, I think it would be beneficial for my patients to incorporate this use of RMT into the way I care for my patients.**

strongly agree / agree / neither agree nor disagree / disagree / strongly disagree

**Overall, I think it would be beneficial for me and the clinical team to incorporate this use of RMT into** **the way we care for patients.**

strongly agree / agree / neither agree nor disagree / disagree / strongly disagree

**Please provide any comments you might have about this use case in the box below.**

|  |
| --- |

**Part 3 – Further considerations**

For the next set of statements, please rate each according to your own experience and views of using RMT. Please indicate whether you **strongly agree, agree, neither agree nor disagree, disagree or strongly disagree** with each one.

**Clinical time**

- Receiving data on a patients’ condition would be an added burden that would not help me to manage their condition.
- Use of RMT by patients would be manageable within my current workload.
- It would take too much time to review data from patients’ RMT.
- Time would be saved in my practice overall if I had access to patients’ RMT data.
- I would not be able to review patients’ data between their consultations.

**Frequency of data collection**

- Mood scores need to be collected from patients on a daily basis, any less frequent collection of data (e.g. weekly) would not be as useful.
- I would review data from my patients between their consultations and I (or a member of my team) would take action in relation to their care between their consultations.
- I would be able to manage my patients’ care better if I had a full year’s worth of data from them (irrespective of cost or practicality of its collection).
- I would only make use of data collected over a period of 1 week prior to a patient’s appointment.

**Technical support**

- I would require a technical helpline that was always available to make best use of RMT data in the care of my patients.
- I would require a training session before starting to make use of RMT data in my practice.
- My patients would require a technical helpline that was always available to them to make best use of RMT.
- Most patients would be able to operate and successfully make use of RMT (given helpline support) to benefit their care.

**Usefulness of data**

- Receiving data on patients’ symptoms more often than it is currently collected would make a significant positive difference to how I manage my patients’ health.
- The costs associated with false positives/negatives would outweigh any potential benefits to their use.
- I think RMT could help patients to know when they need to contact services and when they do not.

**Payment / Reimbursement**

- My clinic would receive less money if the number of face to face appointments per patient was reduced.
- The cost of implementing RMT would be incurred outside of my immediate practice
- It would be difficult to make a business case for introducing RMT in my area.
- My organisation would invest in RMT (spend money to implement RMT) for these use cases
  - In depression
  - In epilepsy
  - In MS

**Rating of concerns around use of RMT**

Our prior work has revealed some concerns around the use of RMT in clinical practice. We are seeking to define which of these would have the greatest impact on uptake.

For the next set of questions, please consider for your clinical context whether each of the stated concerns: i) would not prevent use, ii) would prevent you using RMT in certain situations, or iii) would prevent you from using RMT entirely.

- I have concerns about patients reporting dangers via RMT (e.g. suicidality, SUDEP) and expecting them to be picked up, but healthcare teams not having capacity to respond.
  - would not prevent use / would prevent use in certain situations

/ would prevent use entirely

- I am concerned about the medico-legal consequences of my patients using RMT.
  - would not prevent use / would prevent use in certain situations

/ would prevent use entirely

- I am concerned that patients could rely too much on RMT and would not contact services when they should.
  - would not prevent use / would prevent use in certain situations

/ would prevent use entirely

- I believe RMT would cause patients too much extra anxiety.
  - would not prevent use / would prevent use in certain situations

/ would prevent use entirely

- Interoperability (using a new type of technology alongside existing software and technology) remains a problem with introducing new technologies in health care settings.
  - would not prevent use / would prevent use in certain situations

/ would prevent use entirely

- I believe there are unresolvable issues around information governance in relation to remotely collected data.
  - would not prevent use / would prevent use in certain situations

/ would prevent use entirely

- Some patients may use RMT when they are not engaged in clinical care, or when not appropriate, and this may cause problems.
  - would not prevent use / would prevent use in certain situations

/ would prevent use entirely

**Next steps**

Thank you for completing our survey. Please could you enter your email address so we can contact you to take part in the second part of the survey in around 2 months’ time?

Would you be interested in taking part in a short follow-up interview after the second survey? If so, please tick this box. We would be grateful for your participation.
