# Supplementary material for: Clinical Perspectives on Using Remote Measurement Technology in Assessing Epilepsy, Multiple Sclerosis, and Depression: Delphi Study
Source: JMIR Neurotechnol. 2023 Apr 25;2:e41439. doi: 10.2196/41439 (PMC12671310; doi:10.2196/41439)
Supplement: Multimedia Appendix 3 [file neuro_v2i1e41439_app3.docx]

# Multimedia Appendix 3: Interview guide

The interview guide was modified in preparation for each interview, to include questions relevant to each interviewee’s particular answers given in the survey, in particular where these differed from the majority of responses. The below interview guide was used as a basis for discussion.

*Before starting, check completion and receipt of consent form.*

**Introduction script:**

Thank you for agreeing to take part in this follow-up interview. The purpose of this is to get more of an insight into your views of the use cases presented, and to further explore your thinking when completing the survey. I would like to record the interview so we can make best use of the information you provide, is that ok?

START RECORDING

**Demographics:**

To clarify, based on your survey, I see that you are a [job role] and that you treat patients with [conditions mentioned on their survey], is that correct? Thank you for confirming.

**Main questions:**

1. Firstly, I’d like to ask about your views of the use case diagrams you reviewed in the survey. Did you have any particular thoughts when reviewing these about:
   1. The practicality of implementing RMT in this use case?
   2. Any clear and immediate benefits it would bring you, your patients or the service/trust you work for?

Follow-up questions as appropriate:

*What would need to change to accommodate the introduction of these in your field? What costs would be involved? Extra staff? Staff training? IT support? Would the NHS provide off the shelf devices to permit use of something like this, or would they generally rely on people to have their own – (based on any experience you can draw on - examples of new tech in your own work.)*

1. Secondly, the survey asked about clinical time, frequency of data collection and technical support. Did you have any thought about these areas beyond what was written on the survey?

(Follow-up questions as appropriate)

1. The survey also asked about the usefulness or value of the data that could be generated using RMT, including the impact of false positives and false negatives. Do you have any further views on the value of RMT data and how it might impact the care you give?

(Follow-up questions as appropriate)

1. We also asked you about payment/reimbursement of the use of RMT. Are there any specific factors related to your particular context that would affect the business case or reimbursement of RMT by your health service, for example, any negative impact of reducing face-to-face appointments?

(Follow-up questions as appropriate)

1. Do you have any concerns about the use of RMT with patients living with [condition] that weren’t covered in the survey?
2. Are you aware of any regional or national infrastructural support/incentivisation for digital technology?
3. Who would be the payors in your context? Would payment for these initiatives come out of existing internal budgets, or is there local support, or national support?
4. What are the factors affecting use of RMT in your country and context compared to others you may be aware of?

Thank you very much for your time. I’ll stop recording there.
