# Supplementary material for: Clinical Perspectives on Using Remote Measurement Technology in Assessing Epilepsy, Multiple Sclerosis, and Depression: Delphi Study
Source: JMIR Neurotechnol. 2023 Apr 25;2:e41439. doi: 10.2196/41439 (PMC12671310; doi:10.2196/41439)
Supplement: Multimedia Appendix 5 [file neuro_v2i1e41439_app5.docx]

# Multimedia Appendix 5. Table S1. Results of consensus and stability analyses.

|  | **Round 1 consensus** | | | | | **Round 2 consensus** | | | | | **Stability** | | |
| --- | --- | --- | --- | --- | --- | --- | --- | --- | --- | --- | --- | --- | --- |
| **Question item** | **N responses** | **Median (IQR)** | **Rated 'Agree' or above,  n (%) *** | **Rated 'Disagree' or below,  n (%) *** | **Rated 'Neutral',  n (%) *** | **N responses** | **Median (IQR)** | **Rated 'Agree' or above,  n (%) *** | **Rated 'Disagree' or below,  n (%) *** | **Rated 'Neutral',  n (%) *** | **Agreement between rounds, %** | **Gwet's Agreement Coefficient (95% CI)** | **Benchmark interval (Altman, 1991) **** |
| Ep1 practical | 6 | 4.5 (1) | **6 (100%)** | 0 (0%) | 0 (0%) | 6 | 4.0 (1) | **6 (100%)** | 0 (0%) | 0 (0%) | 98% | 0.97 (0.89 - 1.00) | **0.80 - 1.00** |
| Ep1 enable | 6 | 4.5 (1) | **6 (100%)** | 0 (0%) | 0 (0%) | 6 | 4.0 (1) | **5 (83%)** | 0 (0%) | 1 (17%) | 97% | 0.93 (0.81 - 1.00) | **0.80 - 1.00** |
| Ep1 facilitate | 6 | 4.0 (1) | **6 (100%)** | 0 (0%) | 0 (0%) | 6 | 4.5 (1) | **6 (100%)** | 0 (0%) | 0 (0%) | 98% | 0.97 (0.89 - 1.00) | **0.80 - 1.00** |
| Ep1 enhance | 6 | 4.0 (1) | **6 (100%)** | 0 (0%) | 0 (0%) | 6 | 4.0 (1) | **6 (100%)** | 0 (0%) | 0 (0%) | 97% | 0.94 (0.84 - 1.00) | **0.80 - 1.00** |
| Ep1 catalyse | 6 | 4.0 (1) | 4 (67%) | 0 (0%) | 2 (33%) | 6 | 4.0 (2) | 4 (67%) | 1 (17%) | 1 (17%) | 97% | 0.91 (0.74 - 1.00) | **0.80 - 1.00** |
| Ep1 beneficial patients | 6 | 4.0 (0) | **6 (100%)** | 0 (0%) | 0 (0%) | 6 | 4.0 (1) | **5 (83%)** | 0 (0%) | 1 (17%) | 97% | 0.94 (0.82 - 1.00) | **0.80 - 1.00** |
| Ep1 beneficial clinicians | 6 | 4.0 (1) | **6 (100%)** | 0 (0%) | 0 (0%) | 6 | 4.0 (1) | **5 (83%)** | 0 (0%) | 1 (17%) | 95% | 0.90 (0.77 - 1.00) | **0.80 - 1.00** |
| Ep2 practical | 6 | 5.0 (1) | **6 (100%)** | 0 (0%) | 0 (0%) | 6 | 4.0 (1) | **6 (100%)** | 0 (0%) | 0 (0%) | 97% | 0.94 (0.84 - 1.00) | **0.80 - 1.00** |
| Ep2 enable | 6 | 5.0 (1) | **6 (100%)** | 0 (0%) | 0 (0%) | 6 | 4.0 (1) | **5 (83%)** | 0 (0%) | 1 (17%) | 93% | 0.86 (0.57 - 1.00) | **0.80 - 1.00** |
| Ep2 facilitate | 6 | 5.0 (1) | **6 (100%)** | 0 (0%) | 0 (0%) | 6 | 4.5 (1) | **6 (100%)** | 0 (0%) | 0 (0%) | 95% | 0.91 (0.80 - 1.00) | **0.80 - 1.00** |
| Ep2 enhance | 6 | 5.0 (1) | **6 (100%)** | 0 (0%) | 0 (0%) | 6 | 4.0 (1) | **6 (100%)** | 0 (0%) | 0 (0%) | 97% | 0.94 (0.84 - 1.00) | **0.80 - 1.00** |
| Ep2 catalyse | 6 | 4.5 (2) | 4 (67%) | 0 (0%) | 2 (33%) | 6 | 5.0 (1) | **6 (100%)** | 0 (0%) | 0 (0%) | 88% | 0.76 (0.38 - 1.00) | 0.60 - 0.80 |
| Ep2 beneficial patients | 6 | 4.5 (1) | **6 (100%)** | 0 (0%) | 0 (0%) | 6 | 4.0 (1) | **6 (100%)** | 0 (0%) | 0 (0%) | 98% | 0.97 (0.89 - 1.00) | **0.80 - 1.00** |
| Ep2 beneficial clinicians | 6 | 4.5 (1) | **6 (100%)** | 0 (0%) | 0 (0%) | 6 | 5.0 (1) | **6 (100%)** | 0 (0%) | 0 (0%) | 98% | 0.97 (0.89 - 1.00) | **0.80 - 1.00** |
| Ep3 practical | 6 | 4.0 (1) | **5 (83%)** | 0 (0%) | 1 (17%) | 6 | 4.0 (0) | **5 (83%)** | 0 (0%) | 1 (17%) | 98% | 0.97 (0.87 - 1.00) | **0.80 - 1.00** |
| Ep3 enable | 6 | 5.0 (1) | **5 (83%)** | 0 (0%) | 1 (17%) | 6 | 4.0 (1) | **5 (83%)** | 0 (0%) | 1 (17%) | 97% | 0.93 (0.81 - 1.00) | **0.80 - 1.00** |
| Ep3 facilitate | 6 | 4.5 (1) | **5 (83%)** | 0 (0%) | 1 (17%) | 6 | 4.0 (1) | 4 (67%) | 0 (0%) | 2 (33%) | 95% | 0.88 (0.74 - 1.00) | **0.80 - 1.00** |
| Ep3 enhance | 6 | 4.0 (1) | **5 (83%)** | 0 (0%) | 1 (17%) | 6 | 4.0 (1) | **5 (83%)** | 0 (0%) | 1 (17%) | 100% | 1.00 (1.00 - 1.00) | **0.80 - 1.00** |
| Ep3 catalyse | 6 | 3.5 (1) | 3 (50%) | 0 (0%) | 3 (50%) | 6 | 3.0 (1) | 2 (33%) | 0 (0%) | 4 (67%) | 95% | 0.91 (0.80 - 1.00) | **0.80 - 1.00** |
| Ep3 beneficial p | 6 | 4.0 (1) | **5 (83%)** | 0 (0%) | 1 (17%) | 6 | 4.0 (0) | **5 (83%)** | 0 (0%) | 1 (17%) | 97% | 0.94 (0.82 - 1.00) | **0.80 - 1.00** |
| Ep3 beneficial c | 6 | 4.0 (2) | 4 (67%) | 0 (0%) | 2 (33%) | 6 | 3.5 (1) | 3 (50%) | 1 (17%) | 2 (33%) | 92% | 0.78 (0.42 - 1.00) | 0.60 - 0.80 |
| Ep4 practical | 6 | 4.0 (0) | **5 (83%)** | 1 (17%) | 0 (0%) | 6 | 4.0 (0) | **6 (100%)** | 0 (0%) | 0 (0%) | 95% | 0.92 (0.69 - 1.00) | **0.80 - 1.00** |
| Ep4 enable | 6 | 4.0 (1) | **5 (83%)** | 0 (0%) | 1 (17%) | 6 | 4.0 (0) | **5 (83%)** | 0 (0%) | 1 (17%) | 98% | 0.97 (0.87 - 1.00) | **0.80 - 1.00** |
| Ep4 facilitate | 6 | 4.5 (1) | **5 (83%)** | 0 (0%) | 1 (17%) | 6 | 4.0 (0) | **6 (100%)** | 0 (0%) | 0 (0%) | 95% | 0.90 (0.77 - 1.00) | **0.80 - 1.00** |
| Ep4 enhance | 6 | 4.0 (0) | **5 (83%)** | 0 (0%) | 1 (17%) | 6 | 4.0 (0) | **5 (83%)** | 1 (17%) | 0 (0%) | 97% | 0.95 (0.82 - 1.00) | **0.80 - 1.00** |
| Ep4 catalyse | 6 | 4.0 (0) | **5 (83%)** | 0 (0%) | 1 (17%) | 6 | 4.0 (0) | **6 (100%)** | 0 (0%) | 0 (0%) | 98% | 0.98 (0.92 - 1.00) | **0.80 - 1.00** |
| Ep4 beneficial p | 6 | 4.0 (0) | **5 (83%)** | 1 (17%) | 0 (0%) | 6 | 4.0 (0) | **5 (83%)** | 0 (0%) | 1 (17%) | 97% | 0.95 (0.82 - 1.00) | **0.80 - 1.00** |
| Ep4 beneficial c | 6 | 4.0 (0) | **5 (83%)** | 1 (17%) | 0 (0%) | 6 | 4.0 (0) | **5 (83%)** | 0 (0%) | 1 (17%) | 97% | 0.95 (0.82 - 1.00) | **0.80 - 1.00** |
| Ep5 practical | 6 | 4.0 (1) | **6 (100%)** | 0 (0%) | 0 (0%) | 6 | 4.0 (1) | **6 (100%)** | 0 (0%) | 0 (0%) | 97% | 0.94 (0.84 - 1.00) | **0.80 - 1.00** |
| Ep5 enable | 6 | 4.0 (2) | 4 (67%) | 1 (17%) | 1 (17%) | 6 | 4.0 (1) | **5 (83%)** | 0 (0%) | 1 (17%) | 93% | 0.83 (0.63 - 1.00) | **0.80 - 1.00** |
| Ep5 facilitate | 6 | 4.0 (2) | 4 (67%) | 0 (0%) | 2 (33%) | 6 | 4.0 (1) | **5 (83%)** | 0 (0%) | 1 (17%) | 95% | 0.88 (0.74 - 1.00) | **0.80 - 1.00** |
| Ep5 enhance | 6 | 4.0 (2) | 4 (67%) | 0 (0%) | 2 (33%) | 6 | 4.0 (1) | **6 (100%)** | 0 (0%) | 0 (0%) | 93% | 0.85 (0.71 - 0.99) | **0.80 - 1.00** |
| Ep5 catalyse | 6 | 4.0 (0) | **5 (83%)** | 0 (0%) | 1 (17%) | 6 | 4.0 (1) | **6 (100%)** | 0 (0%) | 0 (0%) | 97% | 0.94 (0.82 - 1.00) | **0.80 - 1.00** |
| Ep5 beneficial p | 6 | 4.0 (2) | 4 (67%) | 0 (0%) | 2 (33%) | 6 | 4.0 (1) | **5 (83%)** | 0 (0%) | 1 (17%) | 95% | 0.88 (0.74 - 1.00) | **0.80 - 1.00** |
| Ep5 beneficial c | 6 | 4.0 (2) | 4 (67%) | 0 (0%) | 2 (33%) | 6 | 4.0 (1) | **5 (83%)** | 0 (0%) | 1 (17%) | 95% | 0.88 (0.74 - 1.00) | **0.80 - 1.00** |
| MS1 practical | 3 | 5.0 (1) | **3 (100%)** | 0 (0%) | 0 (0%) | 3 | 4.0 (2) | 2 (67%) | 0 (0%) | 1 (33%) | 93% | 0.85 (0.38 - 1.00) | **0.80 - 1.00** |
| MS1 enable | 3 | 3.0 (1) | 1 (33%) | 0 (0%) | 2 (67%) | 3 | 4.0 (1) | 2 (67%) | 0 (0%) | 1 (33%) | 97% | 0.94 (0.68 - 1.00) | **0.80 - 1.00** |
| MS1 facilitate | 3 | 4.0 (2) | 2 (67%) | 0 (0%) | 1 (33%) | 3 | 3.0 (3) | 1 (33%) | 1 (33%) | 1 (33%) | 83% | 0.52 (0.00 - 1.00) | 0.40 - 0.60 |
| MS1 enhance | 3 | 5.0 (3) | 2 (67%) | 1 (33%) | 0 (0%) | 3 | 4.0 (3) | 2 (67%) | 1 (33%) | 0 (0%) | 97% | 0.93 (0.57 - 1.00) | **0.80 - 1.00** |
| MS1 catalyse | 3 | 4.0 (2) | 2 (67%) | 1 (33%) | 0 (0%) | 3 | 3.0 (3) | 1 (33%) | 1 (33%) | 1 (33%) | 93% | 0.81 (0.28 - 1.00) | **0.80 - 1.00** |
| MS1 beneficial p | 3 | 5.0 (2) | 2 (67%) | 0 (0%) | 1 (33%) | 3 | 4.0 (3) | 2 (67%) | 1 (33%) | 0 (0%) | 93% | 0.83 (0.19 - 1.00) | **0.80 - 1.00** |
| MS1 beneficial c | 3 | 4.0 (2) | 2 (67%) | 0 (0%) | 1 (33%) | 3 | 4.0 (2) | 2 (67%) | 1 (33%) | 0 (0%) | 93% | 0.83 (0.19 - 1.00) | **0.80 - 1.00** |
| MS2 practical | 3 | 4.0 (1) | **3 (100%)** | 0 (0%) | 0 (0%) | 3 | 4.0 (1) | 2 (67%) | 0 (0%) | 1 (33%) | 93% | 0.88 (0.48 - 1.00) | **0.80 - 1.00** |
| MS2 enable | 3 | 3.0 (3) | 1 (33%) | 1 (33%) | 1 (33%) | 3 | 3.0 (3) | 1 (33%) | 1 (33%) | 1 (33%) | 100% | 1.00 (1.00 - 1.00) | **0.80 - 1.00** |
| MS2 facilitate | 3 | 5.0 (3) | 2 (67%) | 1 (33%) | 0 (0%) | 3 | 4.0 (3) | 2 (67%) | 1 (33%) | 0 (0%) | 97% | 0.93 (0.57 - 1.00) | **0.80 - 1.00** |
| MS2 enhance | 3 | 5.0 (2) | 2 (67%) | 0 (0%) | 1 (33%) | 3 | 3.0 (2) | 1 (33%) | 1 (33%) | 1 (33%) | 83% | 0.52 (0.00 - 1.00) | 0.40 - 0.60 |
| MS2 catalyse | 3 | 3.0 (1) | 1 (33%) | 0 (0%) | 2 (67%) | 3 | 2.0 (2) | 1 (33%) | 2 (67%) | 0 (0%) | 93% | 0.83 (0.47 - 1.00) | **0.80 - 1.00** |
| MS2 beneficial p | 3 | 4.0 (2) | 2 (67%) | 0 (0%) | 1 (33%) | 3 | 4.0 (2) | 2 (67%) | 1 (33%) | 0 (0%) | 93% | 0.83 (0.19 - 1.00) | **0.80 - 1.00** |
| MS2 beneficial c | 3 | 4.0 (2) | 2 (67%) | 0 (0%) | 1 (33%) | 3 | 3.0 (2) | 1 (33%) | 1 (33%) | 1 (33%) | 90% | 0.71 (0.54 - 0.89) | 0.60 - 0.80 |
| MS3 practical | 3 | 4.0 (2) | 2 (67%) | 0 (0%) | 1 (33%) | 3 | 4.0 (1) | 2 (67%) | 0 (0%) | 1 (33%) | 97% | 0.93 (0.57 - 1.00) | **0.80 - 1.00** |
| MS3 enable | 3 | 4.0 (2) | 2 (67%) | 0 (0%) | 1 (33%) | 3 | 3.0 (2) | 1 (33%) | 0 (0%) | 2 (67%) | 97% | 0.93 (0.57 - 1.00) | **0.80 - 1.00** |
| MS3 facilitate | 3 | 3.0 (3) | 1 (33%) | 1 (33%) | 1 (33%) | 3 | 4.0 (2) | 2 (67%) | 1 (33%) | 0 (0%) | 93% | 0.81 (0.28 - 1.00) | **0.80 - 1.00** |
| MS3 enhance | 3 | 4.0 (1) | 2 (67%) | 0 (0%) | 1 (33%) | 3 | 3.0 (2) | 1 (33%) | 1 (33%) | 1 (33%) | 93% | 0.85 (0.38 - 1.00) | **0.80 - 1.00** |
| MS3 catalyse | 3 | 4.0 (2) | 2 (67%) | 0 (0%) | 1 (33%) | 3 | 3.0 (2) | 1 (33%) | 1 (33%) | 1 (33%) | 90% | 0.71 (0.54 - 0.89) | 0.60 - 0.80 |
| MS3 beneficial p | 3 | 4.0 (2) | 2 (67%) | 0 (0%) | 1 (33%) | 3 | 4.0 (2) | 2 (67%) | 1 (33%) | 0 (0%) | 93% | 0.83 (0.19 - 1.00) | **0.80 - 1.00** |
| MS3 beneficial c | 3 | 4.0 (2) | 2 (67%) | 0 (0%) | 1 (33%) | 3 | 4.0 (2) | 2 (67%) | 1 (33%) | 0 (0%) | 93% | 0.83 (0.19 - 1.00) | **0.80 - 1.00** |
| Dep1 practical | 9 | 5.0 (1) | **9 (100%)** | 0 (0%) | 0 (0%) | 9 | 4.0 (1) | **9 (100%)** | 0 (0%) | 0 (0%) | 94% | 0.90 (0.83 - 0.97) | **0.80 - 1.00** |
| Dep1 enable | 9 | 5.0 (1) | **9 (100%)** | 0 (0%) | 0 (0%) | 9 | 5.0 (1) | **9 (100%)** | 0 (0%) | 0 (0%) | 99% | 0.98 (0.94 - 1.00) | **0.80 - 1.00** |
| Dep1 facilitate | 9 | 4.0 (1) | **7 (78%)** | 0 (0%) | 2 (22%) | 9 | 4.0 (0) | **7 (78%)** | 1 (11%) | 1 (11%) | 98% | 0.95 (0.85 - 1.00) | **0.80 - 1.00** |
| Dep1 enhance | 9 | 5.0 (1) | **8 (89%)** | 1 (11%) | 0 (0%) | 9 | 4.0 (1) | **7 (78%)** | 0 (0%) | 2 (22%) | 96% | 0.90 (0.77 - 1.00) | **0.80 - 1.00** |
| Dep1 catalyse | 9 | 4.0 (1) | **9 (100%)** | 0 (0%) | 0 (0%) | 9 | 4.0 (1) | 6 (67%) | 1 (11%) | 2 (22%) | 92% | 0.83 (0.56 - 1.00) | **0.80 - 1.00** |
| Dep1 beneficial p | 9 | 4.0 (1) | **8 (89%)** | 0 (0%) | 1 (11%) | 9 | 4.0 (1) | **8 (89%)** | 0 (0%) | 1 (11%) | 96% | 0.91 (0.81 - 1.00) | **0.80 - 1.00** |
| Dep1 beneficial c | 9 | 4.0 (1) | **9 (100%)** | 0 (0%) | 0 (0%) | 9 | 4.0 (1) | **9 (100%)** | 0 (0%) | 0 (0%) | 98% | 0.96 (0.90 - 1.00) | **0.80 - 1.00** |
| Dep2 practical | 9 | 5.0 (1) | **8 (89%)** | 0 (0%) | 1 (11%) | 9 | 5.0 (1) | **9 (100%)** | 0 (0%) | 0 (0%) | 97% | 0.94 (0.86 - 1.00) | **0.80 - 1.00** |
| Dep2 enable | 9 | 4.0 (0) | **8 (89%)** | 0 (0%) | 1 (11%) | 9 | 4.0 (1) | **8 (89%)** | 0 (0%) | 1 (11%) | 97% | 0.94 (0.85 - 1.00) | **0.80 - 1.00** |
| Dep2 facilitate | 9 | 4.0 (1) | **7 (78%)** | 0 (0%) | 2 (22%) | 9 | 4.0 (0) | **7 (78%)** | 0 (0%) | 2 (22%) | 97% | 0.92 (0.83 - 1.00) | **0.80 - 1.00** |
| Dep2 enhance | 9 | 4.0 (0) | **7 (78%)** | 0 (0%) | 2 (22%) | 9 | 4.0 (0) | **7 (78%)** | 0 (0%) | 2 (22%) | 98% | 0.95 (0.88 - 1.00) | **0.80 - 1.00** |
| Dep2 catalyse | 9 | 4.0 (1) | 5 (56%) | 0 (0%) | 4 (44%) | 9 | 4.0 (0) | **7 (78%)** | 0 (0%) | 2 (22%) | 98% | 0.95 (0.87 - 1.00) | **0.80 - 1.00** |
| Dep2 beneficial p | 9 | 4.0 (0) | **7 (78%)** | 0 (0%) | 2 (22%) | 9 | 4.0 (0) | **7 (78%)** | 0 (0%) | 2 (22%) | 96% | 0.90 (0.81 - 1.00) | **0.80 - 1.00** |
| Dep2 beneficial c | 9 | 4.0 (0) | **7 (78%)** | 0 (0%) | 2 (22%) | 9 | 4.0 (0) | **7 (78%)** | 0 (0%) | 2 (22%) | 96% | 0.90 (0.81 - 1.00) | **0.80 - 1.00** |
| Dep3 Practical | 9 | 5.0 (1) | **8 (89%)** | 1 (11%) | 0 (0%) | 9 | 5.0 (1) | **9 (100%)** | 0 (0%) | 0 (0%) | 94% | 0.89 (0.72 - 1.00) | **0.80 - 1.00** |
| Dep3 enable | 9 | 5.0 (1) | **8 (89%)** | 1 (11%) | 0 (0%) | 9 | 5.0 (1) | **9 (100%)** | 0 (0%) | 0 (0%) | 96% | 0.92 (0.75 - 1.00) | **0.80 - 1.00** |
| Dep3 facilitate | 9 | 4.0 (1) | **8 (89%)** | 1 (11%) | 0 (0%) | 9 | 4.0 (0) | **7 (78%)** | 0 (0%) | 2 (22%) | 96% | 0.91 (0.79 - 1.00) | **0.80 - 1.00** |
| Dep3 enhance | 9 | 4.0 (1) | **8 (89%)** | 1 (11%) | 0 (0%) | 9 | 4.0 (0) | **8 (89%)** | 0 (0%) | 1 (11%) | 98% | 0.96 (0.87 - 1.00) | **0.80 - 1.00** |
| Dep3 catalyse | 9 | 4.0 (0) | **8 (89%)** | 1 (11%) | 0 (0%) | 9 | 4.0 (0) | **7 (78%)** | 1 (11%) | 1 (11%) | 99% | 0.98 (0.92 - 1.00) | **0.80 - 1.00** |
| Dep3 beneficial p | 9 | 4.0 (1) | **8 (89%)** | 0 (0%) | 1 (11%) | 9 | 4.0 (0) | **8 (89%)** | 0 (0%) | 1 (11%) | 97% | 0.94 (0.85 - 1.00) | **0.80 - 1.00** |
| Dep3 beneficial c | 9 | 4.0 (1) | **8 (89%)** | 0 (0%) | 1 (11%) | 9 | 4.0 (0) | **9 (100%)** | 0 (0%) | 0 (0%) | 96% | 0.92 (0.83 - 1.00) | **0.80 - 1.00** |
| Dep4 Practical | 9 | 5.0 (1) | **8 (89%)** | 0 (0%) | 1 (11%) | 9 | 5.0 (1) | **7 (78%)** | 0 (0%) | 2 (22%) | 98% | 0.96 (0.88 - 1.00) | **0.80 - 1.00** |
| Dep4 enable | 9 | 5.0 (1) | **8 (89%)** | 0 (0%) | 1 (11%) | 9 | 4.0 (1) | **8 (89%)** | 0 (0%) | 1 (11%) | 98% | 0.95 (0.88 - 1.00) | **0.80 - 1.00** |
| Dep4 facilitate | 9 | 4.0 (2) | 5 (56%) | 1 (11%) | 3 (33%) | 9 | 4.0 (0) | **7 (78%)** | 0 (0%) | 2 (22%) | 93% | 0.82 (0.59 - 1.00) | **0.80 - 1.00** |
| Dep4 enhance | 9 | 4.0 (2) | 6 (67%) | 1 (11%) | 2 (22%) | 9 | 5.0 (1) | **7 (78%)** | 0 (0%) | 2 (22%) | 96% | 0.89 (0.67 - 1.00) | **0.80 - 1.00** |
| Dep4 catalyse | 9 | 4.0 (2) | 5 (56%) | 1 (11%) | 3 (33%) | 9 | 4.0 (2) | 6 (67%) | 0 (0%) | 3 (33%) | 96% | 0.88 (0.65 - 1.00) | **0.80 - 1.00** |
| Dep4 beneficial p | 9 | 4.0 (2) | 5 (56%) | 0 (0%) | 4 (44%) | 9 | 4.0 (2) | 6 (67%) | 0 (0%) | 3 (33%) | 93% | 0.85 (0.66 - 1.00) | **0.80 - 1.00** |
| Dep4 beneficial c | 9 | 4.0 (2) | 5 (56%) | 0 (0%) | 4 (44%) | 9 | 4.0 (2) | 6 (67%) | 0 (0%) | 3 (33%) | 92% | 0.81 (0.63 - 1.00) | **0.80 - 1.00** |
| Dep5 practical | 9 | 4.0 (1) | **9 (100%)** | 0 (0%) | 0 (0%) | 9 | 4.0 (1) | **8 (89%)** | 0 (0%) | 1 (11%) | 94% | 0.89 (0.80 - 0.98) | **0.80 - 1.00** |
| Dep5 enable | 9 | 5.0 (1) | **8 (89%)** | 0 (0%) | 1 (11%) | 9 | 5.0 (1) | **8 (89%)** | 0 (0%) | 1 (11%) | 96% | 0.91 (0.80 - 1.00) | **0.80 - 1.00** |
| Dep5 facilitate | 9 | 4.0 (1) | **8 (89%)** | 0 (0%) | 1 (11%) | 9 | 4.0 (0) | **7 (78%)** | 0 (0%) | 2 (22%) | 94% | 0.88 (0.68 - 1.00) | **0.80 - 1.00** |
| Dep5 enhance | 9 | 4.0 (1) | **8 (89%)** | 0 (0%) | 1 (11%) | 9 | 4.0 (1) | **7 (78%)** | 0 (0%) | 2 (22%) | 92% | 0.83 (0.56 - 1.00) | **0.80 - 1.00** |
| Dep5 catalyse | 9 | 5.0 (1) | **8 (89%)** | 0 (0%) | 1 (11%) | 9 | 4.0 (1) | **7 (78%)** | 0 (0%) | 2 (22%) | 92% | 0.82 (0.57 - 1.00) | **0.80 - 1.00** |
| Dep5 beneficial p | 9 | 4.0 (1) | **8 (89%)** | 0 (0%) | 1 (11%) | 9 | 4.0 (1) | **7 (78%)** | 0 (0%) | 2 (22%) | 92% | 0.83 (0.56 - 1.00) | **0.80 - 1.00** |
| Dep5 beneficial c | 9 | 4.0 (1) | **8 (89%)** | 0 (0%) | 1 (11%) | 9 | 4.0 (1) | **7 (78%)** | 0 (0%) | 2 (22%) | 92% | 0.83 (0.56 - 1.00) | **0.80 - 1.00** |
| Receiving data on a patients’ condition would be an added burden that would not help me to manage their condition | 17 | 2.0 (1) | 2 (12%) | 10 (59%) | 5 (29%) | 17 | 2.0 (1) | 2 (12%) | **12 (71%)** | 3 (18%) | 91% | 0.79 (0.55 - 1.00) | 0.60 - 0.80 |
| Reviewing patients' RMT data would be manageable within my current workload. | 17 | 4.0 (1) | 9 (53%) | 3 (18%) | 5 (29%) | 17 | 3.0 (1) | 6 (35%) | 4 (24%) | 7 (41%) | 89% | 0.74 (0.39 - 1.00) | 0.60 - 0.80 |
| It would take too much time to review data from patients’ RMT. | 17 | 3.0 (1) | 4 (24%) | 6 (35%) | 7 (41%) | 17 | 3.0 (2) | 5 (29%) | 6 (35%) | 6 (35%) | 93% | 0.82 (0.68 - 0.96) | **0.80 - 1.00** |
| Time would be saved in my practice if I had access to patients’ RMT data. | 17 | 4.0 (1) | 10 (59%) | 3 (18%) | 4 (24%) | 17 | 3.0 (1) | 5 (29%) | 4 (24%) | 8 (47%) | 94% | 0.83 (0.72 - 0.94) | **0.80 - 1.00** |
| I would not be able to review patients’ data between their consultations. | 17 | 3.0 (2) | 6 (35%) | 7 (41%) | 4 (24%) | 17 | 3.0 (1) | 4 (24%) | 8 (47%) | 5 (29%) | 94% | 0.84 (0.72 - 0.96) | **0.80 - 1.00** |
| Mood scores need to be collected from patients at risk of mental health conditions on a daily basis, any less frequent collection of data (e.g. weekly) would not be as useful. | 17 | 3.0 (1) | 2 (12%) | 8 (47%) | 7 (41%) | 17 | 2.0 (1) | 1 (6%) | **12 (71%)** | 4 (24%) | 86% | 0.64 (0.25 - 1.00) | 0.60 - 0.80 |
| I would review data from my patients between their consultations and I (or a member of my team) would take action in relation to their care between their consultations. | 17 | 4.0 (1) | **12 (71%)** | 2 (12%) | 3 (18%) | 17 | 4.0 (0) | **13 (76%)** | 2 (12%) | 2 (12%) | 95% | 0.90 (0.80 - 1.00) | **0.80 - 1.00** |
| I would be able to manage my patients’ care better if I had a full year’s worth of data from them (irrespective of cost). | 17 | 4.0 (0) | **13 (76%)** | 2 (12%) | 2 (12%) | 17 | 4.0 (0) | **13 (76%)** | 1 (6%) | 3 (18%) | 92% | 0.84 (0.63 - 1.00) | **0.80 - 1.00** |
| If patients collected data for a week before their appointment with me, I would not find any further data to be useful. | 17 | 2.0 (1) | 2 (12%) | 11 (65%) | 4 (24%) | 17 | 3.0 (3) | 4 (24%) | 11 (65%) | 2 (12%) | 77% | 0.48 (0.11 - 0.85) | 0.40 - 0.60 |
| I would require a technical helpline that was always available to make best use of RMT data in the care of my patients. | 17 | 4.0 (1) | **12 (71%)** | 2 (12%) | 3 (18%) | 17 | 4.0 (1) | **12 (71%)** | 3 (18%) | 2 (12%) | 94% | 0.84 (0.70 - 0.98) | **0.80 - 1.00** |
| I would require a training session before starting to make use of RMT data in my practice. | 17 | 4.0 (1) | **15 (88%)** | 0 (0%) | 2 (12%) | 17 | 4.0 (1) | **14 (82%)** | 1 (6%) | 2 (12%) | 95% | 0.88 (0.80 - 0.96) | **0.80 - 1.00** |
| My patients would require a technical helpline that was always available to them to make best use of RMT. | 17 | 4.0 (1) | **13 (76%)** | 0 (0%) | 4 (24%) | 17 | 4.0 (1) | **15 (88%)** | 0 (0%) | 2 (12%) | 95% | 0.89 (0.79 - 0.99) | **0.80 - 1.00** |
| Most patients would be able to operate and successfully make use of RMT (given helpline support) to benefit their care. | 17 | 4.0 (0) | **14 (82%)** | 2 (12%) | 1 (6%) | 17 | 4.0 (1) | **12 (71%)** | 4 (24%) | 1 (6%) | 93% | 0.86 (0.74 - 0.97) | **0.80 - 1.00** |
| Receiving data on patients’ symptoms more often than it is currently collected would make a significant positive difference to how I manage my patients’ health. | 17 | 4.0 (0) | **15 (88%)** | 1 (6%) | 1 (6%) | 17 | 4.0 (1) | **14 (82%)** | 1 (6%) | 2 (12%) | 98% | 0.96 (0.92 - 1.00) | **0.80 - 1.00** |
| The costs associated with false positives/negatives created by these use cases would outweigh any potential benefits to their use. | 17 | 3.0 (1) | 1 (6%) | 6 (35%) | 10 (59%) | 17 | 3.0 (2) | 4 (24%) | 7 (41%) | 6 (35%) | 90% | 0.76 (0.40 - 1.00) | 0.60 - 0.80 |
| I think RMT could help patients to know when they need to contact services and when they do not. | 17 | 4.0 (0) | **13 (76%)** | 1 (6%) | 3 (18%) | 17 | 4.0 (1) | **12 (71%)** | 1 (6%) | 4 (24%) | 96% | 0.90 (0.82 - 0.98) | **0.80 - 1.00** |
| My clinic would receive less money if the number of face to face appointments per patient was reduced. | 17 | 2.0 (2) | 5 (29%) | 10 (59%) | 2 (12%) | 17 | 3.0 (2) | 7 (41%) | 9 (53%) | 1 (6%) | 92% | 0.80 (0.65 - 0.94) | 0.60 - 0.80 |
| It would be difficult to make a business case for introducing RMT in my area. | 17 | 3.0 (2) | 5 (29%) | 7 (41%) | 5 (29%) | 17 | 3.0 (0) | 2 (12%) | 5 (29%) | 10 (59%) | 92% | 0.80 (0.67 - 0.93) | **0.80 - 1.00** |
| My organisation would invest in RMT (spend money to implement RMT) for these use cases. | 17 | 3.0 (1) | 5 (29%) | 2 (12%) | 10 (59%) | 17 | 3.0 (1) | 6 (35%) | 3 (18%) | 8 (47%) | 89% | 0.76 (0.61 - 0.91) | 0.60 - 0.80 |
| **Question item** | **N responses** | **Median (IQR)** | **Rated ‘Would not prevent me using RMT with my patients’,  n (%) *** | **Rated 'Would prevent use entirely’, n (%) *** | **Rated 'Would prevent use in some situations',  n (%) *** | **N responses** | **Median (IQR)** | **Rated ‘Would not prevent me using RMT with my patients’,  n (%) *** | **Rated 'Would prevent use entirely’, n (%) *** | **Rated 'Would prevent use in some situations',  n (%) *** | **Agreement between rounds, %** | **Gwet's Agreement Coefficient (95% CI)** | **Benchmark interval (Altman, 1991) **** |
| I have concerns about patients reporting dangers via RMT (e.g. suicidality, SUDEP) and expecting them to be picked up, but healthcare teams not having capacity to respond. | 17 | 2 (0) | 4 (24%) | 1 (6%) | **12 (71%)** | 17 | 2 (1) | 5 (29%) | 1 (6%) | 11 (65%) | 94% | 0.89 (0.76 - 1.00) | **0.80 - 1.00** |
| I am concerned about the medico-legal consequences of my patients using RMT. | 17 | 3 (1) | 7 (41%) | 2 (12%) | 4 (24%) | 17 | 2 (1) | 7 (41%) | 2 (12%) | 8 (47%) | 45% | 0.27 (0.86 - 0.00) | 0.40 - 0.60 |
| I am concerned that patients could rely too much on RMT and would not contact services when they should. | 17 | 2 (1) | 8 (47%) | 0 (0%) | 9 (53%) | 17 | 2 (1) | 5 (29%) | 3 (18%) | 9 (53%) | 84% | 0.66 (0.44 - 0.88) | 0.60 - 0.80 |
| I believe RMT would cause patients too much extra anxiety. | 16 | 2 (0) | 3 (18%) | 1 (6%) | 11 (65%) | 16 | 2 (1) | 6 (35%) | 2 (12%) | 6 (35%) | 85% | 0.70 (0.43 - 0.97) | 0.60 - 0.80 |
| Interoperability (using a new type of technology alongside existing software and technology) remains a problem with introducing new technologies in health care settings. | 16 | 2 (0) | 3 (18%) | 3 (18%) | 9 (53%) | 16 | 2 (1) | 4 (24%) | 3 (18%) | 8 (47%) | 86% | 0.66 (0.20 - 1.00) | 0.60 - 0.80 |
| I believe there are unresolvable issues around information governance in relation to remotely collected data. | 17 | 3 (1) | 8 (47%) | 1 (6%) | 5 (29%) | 17 | 2 (1) | 4 (24%) | 2 (12%) | 8 (47%) | 91% | 0.79 (0.41 - 1.00) | 0.60 - 0.80 |
| Some patients may use RMT when they are not engaged in clinical care, or when not appropriate, and this may cause problems. | 17 | 2 (0) | 3 (18%) | 1 (6%) | **12 (71%)** | 17 | 2 (0.5) | 3 (18%) | 4 (24%) | 9 (53%) | 84% | 0.71 (0.46 - 0.96) | 0.60 - 0.80 |
| * Bold indicates greater than threshold (70%) agreement. ** Bold text identifies question items where Gwet’s AC reached the highest benchmark interval. Interval ranges relate to strength of agreement, and are interpreted as follows: 0.80-1.00: Very good [strength of agreement], 0.60-0.80: Good, 0.40-0.60: Moderate, 0.20-0.40: Fair, <0.20: Poor (Altman, 1991)[29]. | | | | | | | | | | | | | |
