# Supplementary material for: Clinical Perspectives on Using Remote Measurement Technology in Assessing Epilepsy, Multiple Sclerosis, and Depression: Delphi Study
Source: JMIR Neurotechnol. 2023 Apr 25;2:e41439. doi: 10.2196/41439 (PMC12671310; doi:10.2196/41439)
Supplement: Multimedia Appendix 6 [file neuro_v2i1e41439_app6.docx]

# Multimedia Appendix 6. Table S1. Illustrative extracts from interview transcripts relating to each use case.

| Condition/use case | | Extracts from interviews |
| --- | --- | --- |
| **Epilepsy** | | |
|  | Use case 1 (seizure alert) | - “We already have this for some devices.” [Participant 2] - “Clearly as there is a risk of SUDEP with every generalized tonic clonic seizure in the night, you would really want a sensitivity of 100%. You must not miss any ideally, but [...] if that came at the price of a specificity of 70%, then, you know you would get very many false alerts [...] a measure I have found useful when reading the literature is not specificity as such, but the number of false alerts per period [...] I know that if I was woken up twice per night, I would be a zombie during the day and I couldn’t do my job so, or, a false positive rate for such a device of maybe, once a week might be acceptable. And it will also depend on the patient’s risk and the hypothetical parents’ risk tolerance and so on. But like for any screening tool, your sensitivity needs to be super high.” [Participant 9] |
|  | Use case 2 (seizure counting) | - “If the patient forgot the seizure, then you have all the information in this device, so this is a very reliable register.” [Participant 2] - “There’s no way to regularly look at all their seizure diaries and to see if you should do something. This is just not feasible given the number of patients that we care for [...] So the question would be, if there is a way of using artificial intelligence or maybe just simple thresholding of data in order to give you an information that you should look at this.” [Participant 1] |
|  | Use case 3 (risk scoring) | - “And the risk scoring. I think this is difficult. If it was possible then it would be great, but I think this is so personal, so individual that I just think this is very very difficult, to put in practice.” [Participant 2] - “I think there may be medical legal questions that could be a little bit thorny, you know what if the device says today’s low risk, so they go out for a swim with friends. The parents don’t pay attention and they drown. Is it the fault of the device? Is it the fault of the algorithm? Is it your fault for having done the radar study? Is it my fault because I talked to them about the device? You know it’s, I see that as difficult.” [Participant 9] - “I wouldn’t want people to stop doing stuff because they might have a seizure. I mean where would you put the sensitivity and specificity for it to cause more harm than good.” [Participant 4] |
|  | Use case 4 (triage support) | - “It would be great but we don’t have the structure to receive this data and to analyze [...] I suppose, if the technology is there, we should adapt ourselves to the technology and be able to give a response to this data.” [Participant 2] - “Just in terms of workforce, I didn’t think that would necessarily work.” [Participant 9] |
|  | Use case 5 (trend analysis) | - “For 5, trend analysis, yes, this is a very reliable way to register seizures and to see the trend, it could be very, very helpful.” [Participant 2] |
| **Multiple sclerosis** | | |
|  | Use case 1 (detecting silent progression) | - “In my practice I would like to detect the progression of disease. At the moment we have only one medication that is licensed in progressive disease, you know, and so it would be useful to know a bit earlier [...] If you don’t have effective medications, then some people have argued in meetings what is the point of detecting it?” [Participant 19] - “To detect progression, the most useful would be tools to detect gait disorders.” [Participant 3] |
|  | Use case 2 (detecting depression in MS) | - “[Use of RMT in this way] could be useful to open a bit more conversation [with a patient].” [Participant 19] - “It may be that detecting depression would show the development of the disease, but that would not help us so much.” [Participant 3] |
|  | Use case 3 (data donation) | - “That it is very useful to collect this data, so I’ll be interested, [...] but it will not necessarily have a direct impact to my patients.” [Participant 19] - “There are various places we can store data, for example ‘MS Base,’ which is an existing European registry of patient data.” [Participant 3] |
| **Depression** | | |
|  | Use case 1 (detecting trends) | - “Something that is extremely helpful and probably also might enable easier and better recording of patient reported outcomes of patient data. It might save you time doing administrative stuff.” [Participant 11] |
|  | Use case 2 (reviewing treatment) | - “The question is whether that will shape the diagnosis, or whether it will shape the type of treatment that someone prescribes. At least it will give more insight in the behavior of people when they’re depressed, and I think that will be giving pieces of information that you might be using in recurrence prevention plans or things like that [...] I think it could work, but I’m really doubting to say that I’m convinced that it will work because it really depends on how it is implemented in the rest of the treatment.” [Participant 12] |
|  | Use case 3 (self-management) | - “As for depression self management, the nudging part, that of course would be extremely helpful if it works [...] I think that it could work beautifully for some patients.” [Participant 11] |
|  | Use case 4 (comorbid monitoring) | - “We might encounter much more depression if we manage to do this [...] If you look at someone who has a physical disease, for example, having COPD [chronic obstructive pulmonary disorder], that will make you be less energetic and that will make you reduce your activities. And I mean, how can you distinguish that from a depression?” [Participant 12] |
|  | Use case 5 (carer alert) | - “I don’t know whether it is also possible then to change the behavior of the patient.” [Participant 12] - “I think that the carers might not feel responsible enough to be checking the RMT.” [Participant 5] |
